# Global mitigation efforts cannot neglect emerging emitters

**DOI:** 10.1093/nsr/nwac223

**Published:** 2022-10-19

**Authors:** Can Cui, Dabo Guan, Daoping Wang, Jing Meng, Vicky Chemutai, Paul Brenton, Shaohui Zhang, Yuli Shan, Qiang Zhang, Steven J Davis

**Affiliations:** Department of Earth System Science, Tsinghua University, Beijing 100084, China; Department of Earth System Science, Tsinghua University, Beijing 100084, China; The Bartlett School of Sustainable Construction, University College London, London, WC1E 6BT, UK; Department of Computer Science and Technology, University of Cambridge, Cambridge CB3 0FD,UK; Centre for Nature and Climate, World Economic Forum, Geneva CH-1223, Switzerland; School of Urban and Regional Science, Shanghai University of Finance and Economics, Shanghai 200433, China; The Bartlett School of Sustainable Construction, University College London, London, WC1E 6BT, UK; The World Bank, NW Washington, DC 20433, USA; The World Bank, NW Washington, DC 20433, USA; School of Economics and Management, Beihang University, Beijing 100191, China; International Institute for Applied Systems Analysis, Laxenburg 2361, Austria; School of Geography, Earth and Environmental Sciences, University of Birmingham, Birmingham, B15 2TT, UK; Department of Earth System Science, Tsinghua University, Beijing 100084, China; Department of Earth System Science, University of California Irvine, Irvine, CA 92697, USA; Department of Civil and Environmental Engineering, University of California Irvine, Irvine, CA 92697, USA

**Keywords:** emerging emitters, developing countries, climate change mitigation, CO_2_ emissions, net zero

## Abstract

International efforts to avoid dangerous climate change have historically focused on reducing energy-related CO_2_ emissions from countries with either the largest economies (e.g. the EU and the USA) and/or the largest populations (e.g. China and India). However, in recent years, emissions have surged among a different and much less-examined group of countries, raising concerns that a next generation of high-emitting economies will obviate current mitigation targets. Here, we analyse the trends and drivers of emissions in each of the 59 countries where emissions in 2010–2018 grew faster than the global average (excluding China and India), project their emissions under a range of longer-term energy scenarios and estimate the costs of decarbonization pathways. Total emissions from these ‘emerging emitters’ reach as much as 7.5 GtCO_2_/year in the baseline 2.5° scenario—substantially greater than the emissions from these regions in previously published scenarios that would limit warming to 1.5°C or even 2°C. Such unanticipated emissions would in turn require non-emitting energy deployment from all sectors within these emerging emitters, and faster and deeper reductions in emissions from other countries to meet international climate goals. Moreover, the annual costs of keeping emissions at the low level are in many cases 0.2%–4.1% of countries’ gross domestic production, pointing to potential trade-offs with poverty-reduction goals and/or the need for economic support and low-carbon technology transfer from historically high-emitting countries. Our results thus highlight the critical importance of ramping up mitigation efforts in countries that to this point have been largely ignored.

## Teaser

The 59 emerging emitters with faster-than-average growing emissions recently will continue surging the emissions. From costs perspective, the ambitious scenarios to reach the global 1.5°C target are unlikely to be achieved in these countries without substantial further decreases in the costs of non-emitting energy deployment or economic support and low-carbon technology transfer from historically high-emitting countries.

## INTRODUCTION

Fossil-fuel carbon dioxide (CO_2_) emissions are the main cause of global warming. Since the 1990s, analyses of fossil-fuel CO_2_ emissions have focused on a handful of industrialized economies where emissions have been high (the USA [1] and EU [2]) along with populous and rapidly industrializing countries such as China and India [3,4]. Integrated assessment models (IAMs) aggregate the world into regions based on geography and economic development such that low- or middle-income countries with historically small emissions have been typically included in large and undifferentiated groups such as ‘other Africa’ or ‘Rest of World (ROW)’ [5,6]. However, most of the growth in global emissions since 2010 has been among these ‘ROW’ countries. For example, most of the 59 countries whose annual emissions grew faster than the global nations’ average in 2010–2018 (hereinafter ‘emerging emitters’) were developing economies, including many low-income countries [7,8]. Although none of these emerging emitters is individually a large source of emissions today, their combined emissions are greater than any single country except China and the USA, and 65% greater than India's annual emissions in 2018 (the world's third-largest emitter). Thus, the success of international mitigation efforts may hinge upon these emerging emitters and whether their goals of economic growth and human development are achieved using fossil energy.

Here, therefore, we systematically assess recent trends of emissions and their drivers among the 59 emerging emitters (defined as countries whose annual emissions in 2010–2018 grew at or faster than the 2% per year average of all nations, but excluding China and India); project these countries’ future emissions under scenarios that span a range of long-term socio-economic and energy system trajectories; and assess the economic and climate implications of our scenarios. Details of our analytical approach and data sets are provided in the ‘Methods’ section. In summary, we characterize the drivers of each of the 59 countries’ emissions by decomposing fossil-fuel CO_2_ emissions data from the International Energy Agency (IEA) and then disaggregate the countries from the regional groupings in Shared Socio-economic Pathways (SSPs) developed by the MESSAGEix-GAINS IAMs [9–11] and reproject their emissions for the period 2020–2050 based on recent trends of their emissions and energy systems and a range of mitigation efforts. We then evaluate the implications of emissions in our new scenarios for international climate targets and the economic and energy pathways of the emerging emitters.

## EMERGING EMITTERS

**Figure 1.**
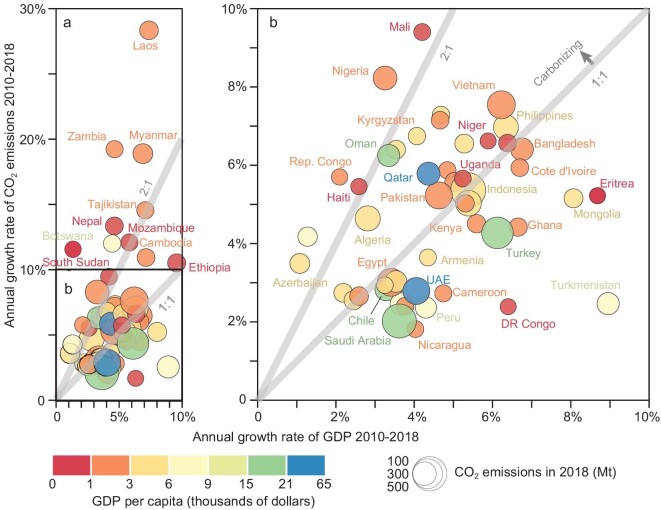
Relative increase in CO_2_ emissions and GDP in 2018 over 2010. Relative increase of GDP and CO_2_ of the 59 countries (due to lack of data, data presented for South Sudan are based on 2012–2015, Eritrea on 2010–2011 and DR Congo on 2010–2017, respectively) with fast-growing emissions in 2018 over levels of 2010 are shown in the panel (a). Each bubble represents a country, plotted by GDP increase in 2018 relative to the level of 2010 on the horizontal and relative CO_2_ emission increase on the vertical. The bubbles of countries with a <100% emission increase (within the black box) in the lower-left part are zoomed in on the right panel (b). The two grey lines with slopes of 1 (lower) and 2 (upper) mean the CO_2_ emission growth rate is the same as or twice the rate of the GDP growth. Countries plotted above the 1:1 line are carbonizing. The size of the bubbles represents the amount of CO_2_ emissions in 2018. The colours represent the per capita GDP of the countries—red for the lower and blue for the higher.

Figure 1 compares the percentage of changes in 2010–2018 in annual CO_2_ emissions and GDP (gross domestic production) among the 59 emerging emitters (listed in Supplementary Table S1; see Supplementary Figs S1 and S2 for emissions by fuel type and by sector). The average annual growth rate of emissions of the 59 countries in 2010–2018 was 6.2%—much higher than the 2.0% average of all nations worldwide, and also higher than the 4.6% annual growth rate of these same countries’ GDP, reflecting increasing use of fossil energy (i.e. carbonization) of their economies. Located in Asia, Africa and Latin America, individually these countries emitted between 0.7 and 542.9 Mt (million tons) of CO_2_ in 2018 (bounded by Eritrea and Indonesia, respectively; Fig. 1). However, together the countries’ annual emissions grew by 40.7% over the period, from 2.7 to 3.8 GtCO_2_ (gigatons of CO_2_). In comparison, emissions in China, the USA and India were 9.6, 4.9 and 2.3 GtCO_2_ in 2018. Moreover, the 1.1-Gt increase in emissions accounts for 38.9% of the global increase in emissions over the period.

The emerging emitters include countries in development categories ranging from the least-developed country to economy in transition (EIT) [8], but in most cases with GDP per capita substantially less than the global average (in 53 of the 59 countries, per capita GDP was <${\$}$11 000/yr in 2018 (constant 2010 USD)). In 2017 the countries were also home to 698 million people in absolute poverty (e.g. <1.9 US${\$}$ per day in purchasing power parity value)—9.3% of global population in that year [12]. Among the 59 countries, emissions grew faster than GDP in 34 (58%) and *twice* as fast as GDP in 12 of these (20%; Fig. 1). In 25 others (42%), economic growth outstripped emissions growth, corresponding to decreasing carbon intensity of those economies.

## DRIVERS OF RECENT EMISSIONS SURGE

**Figure 2.**
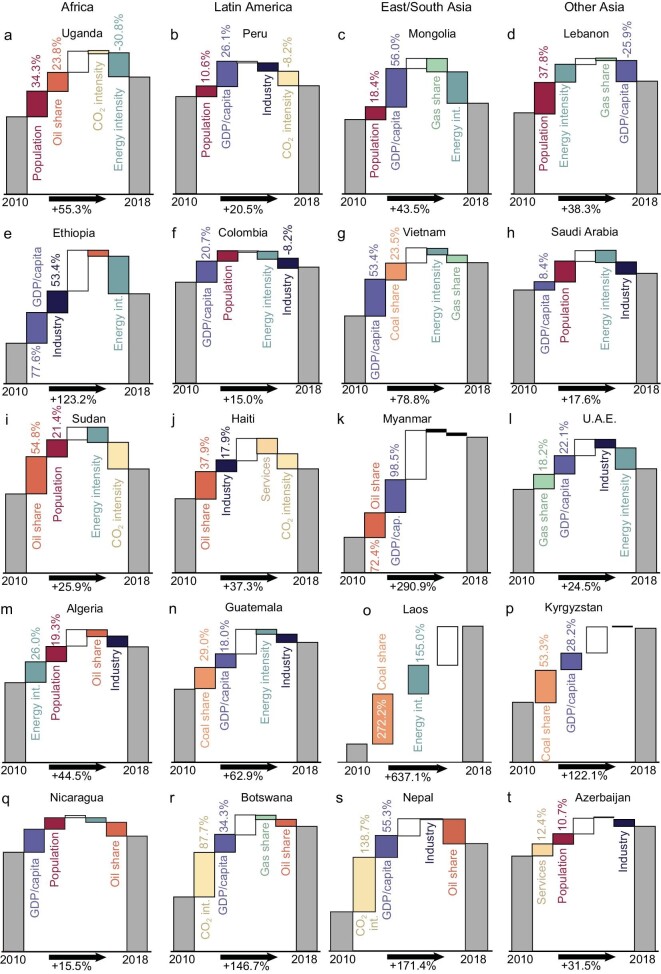
(a–t) Case countries with surging emissions and their drivers. The waterfalls show the drivers of emission growth over 2010–2018, including drivers increasing consumption (population, GDP per capita), drivers affecting economic structure (industrial structure, i.e. share of the value added of primary, secondary and tertiary industry, and the energy intensity of GDP) and drivers affecting carbon intensity (energy structure, i.e. share of consumption of coal, oil, natural gas and other fuel, and CO_2_ emission intensity of energy). Each country has their two main drivers and two main inhibitors shown, and the relative change of emission increased from the level of 2010 to that of 2018.

Figure 2 shows the drivers of changes in emissions in 2010–2018 for 20 emerging emitters in Africa, Latin America and Asia. Supplementary Figs S3–S6 show analogous plots for the other 39 countries. In each case, we plot the two most influential drivers of changes in emissions over the 8-year time period. Across all 59 emerging emitters, population growth (red bars) is most important in 17 (29%) of the countries including Uganda (Fig. 2a) and Lebanon (Fig. 2d), though increases in GDP per capita (dark blue) are the most important factors underlying emission increases in 26 (44%) of the countries including Ethiopia, Colombia and Vietnam (Fig. 2e–g). Following closely behind these socio-economic factors are increases in the use of a particular fossil fuel; increases in either oil (orange) or coal (light orange) are the main drivers of emission increases in 8 of the 59 countries (14%), including Sudan, Haiti, Myanmar, Guatemala and Kyrgyzstan (Fig. 2i, j, k, b and n, respectively; for emissions by fuel type, see Supplementary Figure S3). Energy-intensity (turquoise) increases drove seven (12%) of the countries’ emissions growth as the top-two drivers, including Algeria and Laos (Fig. 2m and o). Increases in the CO_2_ intensity of energy use were also the key drivers of emission increases in a handful (three, or 5%) of the countries, including Nicaragua, Botswana and Nepal (Fig. 2q–s, respectively). Less commonly, increases in the share of value added in GDP represented by industry (black) were also important, such as in Ethiopia (Fig. 2e) and Haiti (Fig. 2j).

In contrast, the factors most important to suppressing the growth of emissions in these countries are declining energy intensity (in 19, or 32%), decreases in CO_2_ intensity of energy use (in 12, or 20% of the countries), decreases in the share of value added by industry (in 9, or 15%, including many Latin American and Other Asian; Fig. 2f, n, h and t) and decreases in the share of oil energy (in 9, or 15%, including Botswana and Nepal). We describe the drivers of case countries’ emissions in greater detail in the Supplementary Information and Supplementary Figs S7–S12.

## PROJECTIONS OF FUTURE EMISSIONS

**Figure 3.**
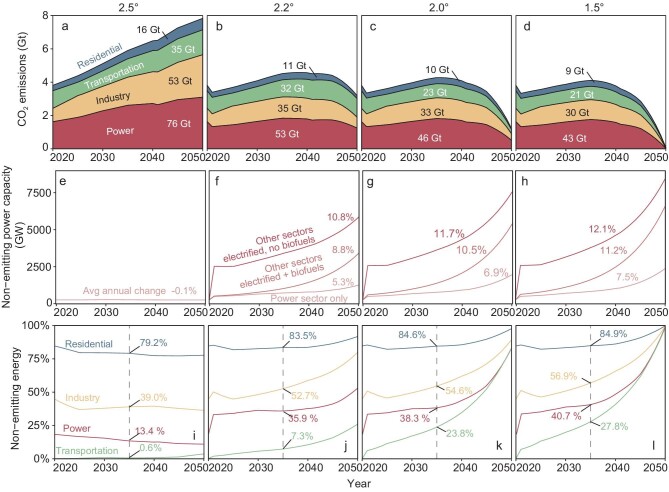
Projected emissions, non-emitting power capacity and non-emitting energy share of sectors and scenarios. Emissions projections under the scenarios of 2.5° baseline, 2.2°, 2.0° and 1.5° are shown in (a–d). Non-emitting power capacity projections of power sector only, and all sector demand are shown in (e–h). Non-emitting energy shares in total energy demand of residential, industry, power and transportation are shown in (i–l) with numbers representing the shares in 2035.

Among the 59 emerging emitters, a major policy priority is economic development to increase incomes and reduce poverty. In turn, GDP per capita is routinely a key driver of emission increases (Fig. 2). Without offsetting decreases in the carbon intensity of these countries’ economies, such development can therefore be expected to spur future growth of emissions. Inertia of emissions from new and historically long-lived energy infrastructures is also a factor in many of these countries [13]. Long-term trajectories of emissions of the emerging emitters will thus be determined by development and energy pathways, and especially of fossil-fuel-based power, industry, transportation and residential sectors. Figure 3 shows the total emissions and shares of non-emitting energy sources from all emerging emitters under a range of scenarios (Fig. 3a; Supplementary Data S2). In each case, these projections include four sector groupings (power, industry, transportation and residential sectors) and assume that the share of non-emitting energy used in each sector (i.e. solar, wind or nuclear energy sources) increases at different rates. Specifically, the 2.5° scenario assumes no deployment of non-emitting energy in 2020–2050 (Fig. 3e; consistent with regional projection by the GAINS model for the RCP4.5 pathway and New Policy Scenario) and would put the world on track to warm 2–3° by 2100 if other countries around the world follow the same pathway; the 2.2° scenario assumes that the capacity non-emitting energy sources grow by 8.8%/year and track toward 2–2.4° of warming by 2100; the 2° scenario assumes 10.5%/year growth in non-emitting energy sources and is consistent with the goal of limiting 2° of warming in 2100; and the 1.5° scenario assumes that the deployment of non-emitting energy increases of 11.2%/year is consistent with the goal of limiting 1.5° of warming in 2100. These scenarios thus span a wide range of mitigation efforts among emerging emitters, from zero to aggressive deployment of low-carbon energy technologies in all sectors (see ‘Methods’ section).

Under the 2.5° scenario, total emissions from the 59 emerging emitters continue rising and reach 7.8 Gt in 2050, with cumulative emissions of 180 GtCO_2_ in 2020–2050 (Fig. 3a). In the other three scenarios, emissions from emerging emitters increase in 2020–2035 and then decline to reach varying levels by 2050. Emissions are 3.6, 1.2 and 0.1 GtCO_2_/year in 2050 in the 2.2°, 2° and 1.5° scenarios, respectively, reflecting reductions relative to the 2.5° scenario of 54.1%, 84.2% and 98.8%, respectively. In all scenarios, the power sector is the largest contributor to cumulative emissions (40.6%–42.2%), followed by industry (26.8%–29.4%), transportation (19.4%–24.5%) and residential (8.4%–8.9%) sectors (Fig. 3a–d).

Scenarios with greater emission reductions correspond to those with more rapid deployment of non-emitting energy technologies (e.g. renewable generation, nuclear power and biofuels) among emerging emitters. In the 2.5° scenario, non-emitting power capacity remains nearly constant at 2020 levels (average annual change in 2020–2050 of –0.1%; Fig. 3e). In the mitigation scenarios, non-emitting power capacity increases by an average of 5.3%, 6.9% and 7.5% annually, but this rate increases considerably if industry, transportation and residential energy demand is also electrified, to as high as 11%–12% in the 2° and 1.5° scenarios and depending on the availability of biofuels (darker red curves in Fig. 3g and h; in cases with biofuels, the energy mix of electricity and biofuels economy-wide is held constant at 2019 levels). Whether by electricity or carbon-neutral biofuels, large shares of residential, industrial and transportation energy come from non-emitting sources by 2050 in the 2.2°, 2° and 1.5° scenarios (Fig. 3j–l), especially in the industry and transportation sectors (residential sector in emerging emitters was already dominated by biofuels in 2018). In the 1.5° scenario, the share of non-emitting energy reaches 100% for all sectors by 2050.

**Figure 4.**
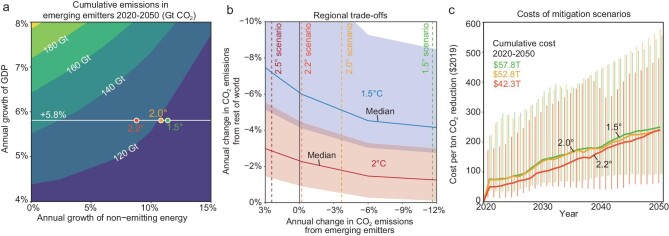
Comparisons of emerging emitter scenarios and climate targets. (a) Cumulative CO_2_ emissions from emerging emitters in 2020–2050 (contours) depend upon both energy demand of economic growth and the non-emitting energy growth. The white line shows the annual growth rate of GDP under the scenarios, and the red, orange and yellow points show the 2.2°, 2° and 1.5° scenarios. (b) Annual changes in emissions from emerging emitters have important implications for the rate of emission reductions required in the rest of the world to limit global warming to below 1.5°C or 2°C. The blue line shows the combinations of the annual changes of emissions of the two types of countries to reach the 1.5° target, and the shadow shows the 66% confidence interval for that. The red line and shadow represent the same for the 2° target. Vertical dashed lines represent the annual growth rate of the emerging emitters under the scenarios of the 2.5°, 2.2°, 2° and 1.5° scenarios. (c) Costs per ton of CO_2_ reduction and cumulative costs of 2.2° (red), 2° (orange) and 1.5° (yellow) scenarios. Error bars represent the 66% confidence intervals of costs per ton of CO_2_ reduction of corresponding scenarios.

Figure 4a represents cumulative emissions from emerging emitters in 2020–2050 as a function of annual growth in the countries’ GDP versus annual growth in non-emitting sources of energy (Fig. 4a). For example, if GDP among emerging emitters grows at 5.8% per year as projected in SSP2-4.5, cumulative emissions in 2020–2050 are 180 GtCO_2_ in the 2.5° scenario, decreasing to 131, 112 and 103 GtCO_2_ in the 2.2°, 2° and 1.5° scenarios, respectively (red, yellow and green circles in Fig. 4a). But the temperatures assigned to our scenarios assume that the rest of the world is mitigating accordingly, and there are trade-offs between emerging emitters and countries in the rest of the world if that is not the case (Supplementary Fig. S13). Figure 4b shows how much faster countries in the rest of the world would need to reduce their emissions depending on what path emerging emitters follow. For example, if emerging emitters follow our 2.5° scenario (i.e. their emissions increase by 2.4%/year), stabilizing global temperatures at 1.5° would require countries in the rest of the world to decarbonize at 7.2%/year (at the median of budgets [14]), as opposed to 4.2%/year if emerging emitters are also on the 1.5° scenario (i.e. their emissions decrease by 11.6%/year; Fig. 4b). Yet the decarbonization rates required in other countries to meet international climate targets are not particularly sensitive to changes in the rate of decarbonization in emerging emitters beyond ∼6%/year; cumulative emissions from emerging emitters are very similar under the 2° and 1.5° scenarios and in both cases are small compared to global budgets (Fig. 4a).

Using projected costs from the literature [15], we estimate that the annual costs of keeping emissions at the 1.5° scenario are in many cases 0.2%–4.1% of countries’ GDP, and the cost of non-emitting sources of electricity (i.e. renewable or nuclear generation) in our 1.5° scenario represents a median of 4.9% of projected annual GDP of emerging emitters in 2050 (1.2%–14.4%; *P* < 0.05; see Supplementary Fig. S14); for the 2° scenario, the share decreases slightly to 4.4% (1.1%–13.6%; *P* < 0.05). For example, the median cost of replacing fossil fuels with non-emitting energy would cost Ethiopia 11.0% of its GDP in 2050. Costs per ton of emissions avoided increase from ${\$}$240.3 per tCO_2_ in the 2.2° scenario to ${\$}$239.2 and ${\$}$249.9 per tCO_2_ under the 2.0° and 1.5° scenarios, respectively, with cumulative costs in 2020–2050 of ${\$}$42.3–57.8 trillion (Fig. 4c), i.e. 0.6%–0.8% of the global GDP over the period. These costs can be paid domestically or from financial transfers from high-income regions.

## DISCUSSION AND CONCLUSIONS

None of the countries we identify as emerging emitters have emitted >2% of global emissions in recent years, but together they have dominated the growth of such emissions over the past decade and will have an important influence on cumulative fossil emissions this century. In particular, as these countries recover from the Covid pandemic, their economic development and investments in energy infrastructure are likely to set the carbon intensity of their economies for decades to come [16]. Indeed, our results suggest that the longer-term trajectories of emissions will depend upon climate and energy policies as economic growth in these countries resumes. Yet sustained economic growth, crucial for poverty reduction, as well as projected increases in population will continue to drive growth in CO_2_ emissions in these countries, even assuming rapid deployment of non-emitting energy across all sectors. Given their importance and unique circumstances, future projections and energy-emission models would do well to disaggregate ‘ROW’ regions and resolve country-specific pathways.

Reductions in these countries’ future emissions depend on rapid deployment of non-emitting energy in all sectors. Yet we have shown that the benefit of increased ambition among emitters has diminishing returns in our scenarios; the rate of emission reductions required in other countries of the world is reduced much more by shifting emerging emitters from a 2.5° scenario to a 2.0° scenario than it is by shifting from a 2.0° to a 1.5° scenario. Moreover, the costs of ambitious mitigation are large, representing >4% of projected GDP in 2050. But these costs must be compared to the costs of instead meeting rising energy demand with fossil-fuel energy, as well as the cost per ton of emissions avoided in other regions, e.g. the annual GDP loss to reach 1.5°C-target emissions in Japan is estimated to be as high as 4.5% [17]. Making such comparisons suggests that, although daunting, mitigation among emerging emitters may be cost-effective, and further makes the case for economic support and technology transfer from higher-income countries on the basis of both human development and meeting climate goals.

## METHODS

Detailed methods and materials are given in the online Supplementary Materials.

## DATA AVAILABILITY

Historical data for 2010–2018: CO_2_ emissions from fuel combustions and energy consumption data over 2010–2018 are from the IEA [7,18], covering data of >140 countries by energy type and economic sector. The population and the GDP data, and the industrial structure data, i.e. the percentage of agriculture, forestry and fishing, industry and services in value added, are from the World Bank [19]. Data for 2019–2050: the CO_2_ emissions data for 2019 are extrapolated based on data for 2010–2018, and the CO_2_ emissions for 2020 are collected from the work of Le Quéré *et al.* Nature Climate Change (2020) [20]. The BAU assumption data including energy mix data and CO_2_ emissions data are from the ‘No Policies Scenario’ projections of the GAINS model from IIASA that developed them based on IEA’s world energy outlook 2018 [9]. The CO_2_ emissions data, the population data and the GDP data under SSPs are from the SSP Database (version 2.0) [21–26]. Data for global warming of 1.5°C and 2°C: the CO_2_ emissions data of countries under the 1.5°C and 2°C global-warming scenarios are compiled from the Integrated Assessment Modeling Consortium (IAMC) 1.5°C Scenario [27]. The used data include the CO_2_ emissions of the world under 1.5°C and 2°C scenarios. The comparison between the projections of this work and the SSPs data is shown in Supplementary Fig. S14 and Supplementary Data S2. All data are available in the main text or the Supplementary information. All the codes associated with this paper are available from the corresponding authors upon reasonable request.

## Supplementary Material

nwac223_Supplemental_Files
