# Supplementary material for: Global mitigation efforts cannot neglect emerging emitters
Source: Natl Sci Rev. 2022 Oct 19;9(12):nwac223. doi: 10.1093/nsr/nwac223 (PMC9757683; doi:10.1093/nsr/nwac223)
Supplement: nwac223_Supplemental_Files [file nwac223_supplemental_files.zip › Supplemental_Information_2022Oct.docx]

Supplemental Information for

**Global mitigation efforts cannot neglect emerging emitters**

Can Cui, Dabo Guan^*^, Daoping Wang, Jing Meng, Vicky Chemutai, Paul Brenton, Shaohui Zhang, Yuli Shan, Qiang Zhang, Steven J. Davis

Correspondence to: guandabo@tsinghua.edu.cn

**This PDF file includes:**

Methods and Materials

Figs. S1 to S15

Captions for Table S1 to S2

**Other Supplementary Materials for this manuscript include the following:**

Table S1 to S2 [The 59 countries with fast-growing CO_2_ emissions and their developing stage; CO_2_ emissions of different scenarios over 2020-2050].

Methods and Materials

Emission drivers: index decomposition analysis.

We divide the emissions growth (C) over 2010 to 2018 into contributions of six drivers: C_P_ from population (P) growth; C_G_ from economic growth measured by GDP per capita (GPC); C_IS_ from industrial structure (IS), as the share of GDP of primary industry, secondary industry, and tertiary industry; C_EI_ from energy intensity (EI) that is energy consumption (E) per unit of GDP; C_ES_ from energy structure (ES), as the share of consumption of energy types including coal, oil, natural gas, and other types; and C_CI_ from CO_2_ emissions intensity (CI) that is emissions per unit of energy consumption, as follows:

$$C=\sum_{ij} P\times\frac{G}{P}\times\frac{G_{i}}{G}\times\frac{E_{i}}{G_{i}}\times\frac{E_{ij}}{E_{i}}\times\frac{C_{ij}}{E_{ij}}=\sum_{ij} P\times GPC\times{IS}_{i}\times{EI}_{i}\times{ES}_{ij}\times{CI}_{ij}$$

Where, *i* refers to the *i*th industry in primary industry, secondary industry, and tertiary industry; *j* refers to the *j*th energy type in coal, oil, natural gas, and other types. The change in C from time 0 to time T can be divided into six parts using logarithmic mean Divisa index (LMDI) method[1,2] as follows:

$$\Delta C=C^{T}-C^{0}=\Delta C_{P}+\Delta C_{GPC}+\Delta C_{IS}+\Delta C_{EI}+\Delta C_{ES}+\Delta C_{CI}$$

where:

$$\Delta C_{X}=\sum_{ij} \frac{C_{ij}^{T}-C_{ij}^{0}}{\ln C_{ij}^{T}-\ln C_{ij}^{0}}\times\ln\left( \frac{X_{ij}^{T}}{X_{ij}^{0}} \right)$$

where, *X_ij_* refers to the driving factors, i.e. *P*, *GPC*, *IS_i_*, *EI_i_*, *ES_ij_*, and *CI_ij_*.

Emission scenario settings for over 2020-2050.

In each case, these projections include four sector groupings (power, industry, residential and transportation) and assume that energy demand in each sector becomes non-emitting at certain annual growth. Specifically, we develop four scenarios for each sector grouping: a 2.5° baseline scenario that assumes no deployment of non-emitting energy over 2020-2050, i.e., the emitters follow the RCP 4.5 and SSP2 pathway of development and reach the 2050 emission target of global warming of 2~3 degree by 2100; a “2.2° ambition” scenario, in which the deployment of non-emitting energy grows by 8.8% annually over the period and reach the 2050 emission target of global warming of 2~2.4 degree by 2100; a “2-degree ambition” scenario, in which the deployment of non-emitting energy grows by 10.5% annually over the period and reach the 2050 emission target of global warming below 2 degree by 2100; and a “1.5° ambition” scenario that assumes the deployment of non-emitting energy grows by 11.2% annually over the period and reach the 2050 emission target of global warming below 1.5 degree by 2100.

Emissions accounting over 2020-2050

*Country-level CO_2_ emissions trend for three scenarios in 2020-2050.* We selected the SSP2 baseline scenario (middle of the road) with no new policy consideration developed by IIASA’s MESSAGEix[3] and imported into GAINS model(based on the default storyline of SSP2_45 of GAINS)[4–6] to provide CO_2_ emissions and energy mix projections for the 59 emerging emitters for our ‘BAU’ scenario. However, the GAINS model only covers major countries and regions, while more than half of emerging emitters studied here are aggregated into ‘Other Regions’. Among 59 emerging emitters, there are 24 countries’ SSPs are available in GAINS database. For 35 out of 59 countries that future socioeconomic development trajectories are not available, thus, the downscaling and appropriate calibration approaches are used to project country-level emissions, according to the region-level emissions. We present all projected emission data 2020-2050 in Supplementary Table S2. We also provide full comparison between our calibrated data and direct downscaling data from SSPs produced by different IAMs (see Supplementary Fig. S15).

We conduct the following procedures to calibrate the historical CO_2_ emissions and emissions after 2020. As shown in Equation 1, scenario settings (by sector and by different low carbon technology deployment) are applied to GAINS regional energy consumption to get emissions of region *r* under scenario *base* (${Em}_{t,r,base}$). The ratio of ${Em}_{t,r,base}$ to ${Em}_{2020,r,base}$ shows a relative change of year *t* to 2020 under scenario *base*. Applying that relative change to the country historical emissions (${Em}_{2020,c,history}$) and we get the downscaled and calibrated emissions projected of country *c* and scenario *base*. By similar way we obtain the emissions trajectories of 1.5° and 2° targets.

| ${Em}_{t,c,base}={Em}_{2020,c,history}\times\frac{{Em}_{t,r,base}}{{Em}_{2020,r,base}}$ | Equation 1 |
| --- | --- |

The emissions under the 2.2° ambition, 2° ambition, and 1.5° ambition scenarios are modified based on the sectoral energy demand and non-emitting energy deployment of each designed scenario. For scenario *s*, the emissions of sector *i* in year *t* are calculated by the fossil fuel demand, ${Ene}_{ff,i,t,s}$, and corresponding emission factors, ${EF}_{ff}$. With the non-emitting energy *j* (which can be renewable energy or nuclear) growing at an annual growth rate of $g_{i,j}$, the fossil energy demand gets substituted by non-emitting energy. Therefore, the total emissions at thin year are:

| ${Em}_{t,s}=\sum_{i} {Em}_{i,t,s}=\sum_{i} {Ene}_{ff,i,t,s}\times{EF}_{ff}=\sum_{i} \left( {Ene}_{i,t}-\sum_{j} {Ene}_{i,j,t,s} \right)\times{EF}_{ff}=\sum_{i} \left[ {Ene}_{i,t}-\sum_{j} {Ene}_{i,j,2021,s}\times\left( 1+g_{i,j,s} \right)^{t-2021} \right]\times{EF}_{ff}$ | Equation 2 |
| --- | --- |

Where the ${Ene}_{i,t}$ represents the energy demand in sector *i* in year *t*, ${Ene}_{i,j,2021,s}$ is the non-emitting energy of sector *i* in 2021 under assumptions of scenario *s*. ${Ene}_{i,j,2021,s}$ is set by the newly added fossil energy demand in 2021, while if that is zero or negative, ${Ene}_{i,j,2021,s}$ is determined based on the targeted fossil energy in 2050 and the average $g_{i,j,s}$ for scenario *s*:

| ${Ene}_{i,j,2021,s}=\left\{ \begin{aligned} {Ene}_{ff,i,2021}-{Ene}_{ff,i,2020} , {Ene}_{ff,i,2021}>{Ene}_{ff,i,2020} \\ \frac{{Ene}_{i,2050}-{Ene}_{ff,i,2050,s}}{\left( 1+\bar{g_{i,j,s}} \right)^{29}}, {Ene}_{ff,i,2021}\leq{Ene}_{ff,i,2020} \end{aligned} \right.$ | Equation 3 |
| --- | --- |

Since non-emitting energy refers to biofuels combustion and renewable and nuclear power, the demand for non-emitting power capacity is composed of that from the power sector only and all sector demand. Therefore, Fig.3e-3h show the non-emitting power demand of the power sector only, the non-emitting power demand of all sectors electrified (but biofuels also deployed as non-emitting energy), and the non-emitting power demand of all sectors using non-emitting power without biofuels.

Economic costs estimation for four scenarios

We estimated the economic costs of the deployment of CCS and renewable energy for the power sector in emerging emitters for the 2.2° ambition, 2-degree ambition, and 1.5° ambition scenarios. For renewable energy and nuclear power generation technology, the technology cost is forecast[7] using the Stochastic Exponent Method as:

| $y_{t+1}=y_{t}\left( \frac{X_{t+1}}{X_{t}} \right)^{{-W}_{t+1}}$ | Equation 4 |
| --- | --- |

Where, $y_{t}$ is the technology cost in year $t$, $X_{t}$ is cumulative production and $W$ is the Wright exponent (or learning exponent). In this case, the compound average annual growth rate (CAAGR) of global cumulative electricity generation over the most 5 years is used to specify a future deployment scenario for 2020-2030 projection, except for the PV and wind 2030 forecasts, where we used the CAAGR of cumulative electricity generation in China observed over the most recent 5 years instead. While for 2030-2050 projection, the CAAGR of cumulative electricity generation in China to reach carbon neutrality is used. This method implicitly assumes that deployment, R&D funding, and other variables continue on their recent historical trajectories for the entire duration of the forecasting period.

Renewable energy and nuclear energy for power generation is assumed in the four scenarios for non-thermal power dominated countries. Since the non-fossil energy generation takes a tiny percentage in the global market, in the four scenarios, we assume the costs of renewable and nuclear energy generation are identical across the world, and the deployment grows at the rate of that in China to reach carbon neutrality by 2060. Based on the projected costs of six types ($r$, including wind, solar, geothermal, biomass power, hydropower and nuclear) of renewable and nuclear energy for power generation per kWh per year ($y_{i,t,r}$), the total costs of renewable and nuclear energy for power generation for country $i$ under scenario $s$ in year $t$ are the sum of the costs of the newly built power units, ${Cost\_REN\_new}_{i,s,t}$, and that of the existed ones, ${Cost\_REN\_existed}_{i,s,t}$:

| ${Cost\_REN}_{i,s,t}={Cost\_REN\_new}_{i,s,t}+{Cost\_REN\_existed}_{i,s,t}$ | Equation 5 |
| --- | --- |

Suppose the renewable and nuclear power units generate equal power each year, the annual operating costs remain the same as that of the first year, $Cost\_REN\_new$. The existing power units' costs, ${Cost\_REN\_existed}_{i,s,t0}$, is the sum of the annual operating costs of all the formerly built units (Equation 5). Since the lifecycle of renewable and nuclear power units is usually longer than 20 years, the renewable power units remain in operation during 2020-2050.

| ${Cost\_REN\_existed}_{i,s,t0}=\sum_{t<t0} {Cost\_REN\_new}_{i,s,t}$ | Equation 6 |
| --- | --- |

Newly added energy capacity is partly or fully supplied by renewable and nuclear energy generation. The costs are product of per unit cost $y_{i,t,r}$ and the power generation. By assuming the renewable energy mix as the same as that in 2019 ($\frac{{Ene\_REN}_{2019,r}}{{Ene\_REN}_{2019}}$), using the newly added energy capacity ${Ene\_new}_{i,s,t}$, and the deployment of renewable energy generation ${Deployment}_{i,s,t}$, the total costs of the newly added renewable power generation can be estimated as:

| ${Cost\_REN\_new}_{i,s,t}=\sum_{r} y_{i,t,r}\times\frac{{Ene\_REN}_{2019,r}}{{Ene\_REN}_{2019}}\times{Ene\_new}_{i,s,t}\times{Deployment}_{i,s,t}$ | Equation 7 |
| --- | --- |

Historical data 2010-2018.

CO_2_ emissions from fuel combustions and energy consumption data over 2010-2018 are from the International Energy Agency (IEA)[8,9], covering data of over 140 countries by energy type and economic sector. The population and the GDP data, and the industrial structure data, i.e. the percentage of agriculture, forestry, and fishing, industry and services in value added are from the World Bank[10].

Data for 2019-2050.

The CO_2_ emissions data 2019 are linearly extrapolated based on data 2010-2018, and the CO_2_ emissions 2020 are collected from work of Le Quéré et al. Nature Climate Change (2020)[11]. The BAU assumption data including energy mix data and CO_2_ emissions data are from the “New Policies Scenario” (NPS) projections of GAINS model from IIASA[4]. The CO_2_ emissions data, the population data, and the GDP data under SSPs are from the SSP Database (version 2.0)[12–17].

Data for global warming of 1.5°C and 2°C.

The CO_2_ emissions data of countries under the 1.5°C and 2°C global warming scenarios are compiled from the Integrated Assessment Modeling Consortium (IAMC) 1.5°C Scenario[18]. The used data include the CO_2_ emissions of the world under 1.5°C and 2°C scenarios. For the emissions of the 59 emerging emitters, we use the emissions under the BAU scenario. The emissions of the rest of the world are defined as the emissions of the world minus that of the 59 emerging emitters.

CO_2_ emissions by energy and industry of the emerging emitters

Energy-related drivers contributed largely to the emissions growth of the emerging emitters. The CO_2_ emissions by fuel type and industry from 2010 to 2018 are shown in Fig. S1 (absolute emissions). Over 2010-2018, among the 34 countries that use coal, 23 countries show a rising share of coal-related CO_2_ emissions, and 29 countries increased the absolute emissions from coal consumption. The carbonizing energy mix of the countries should be noticed since it would continue contributing large emissions in the future, if there is no thorough transition into renewable energy.


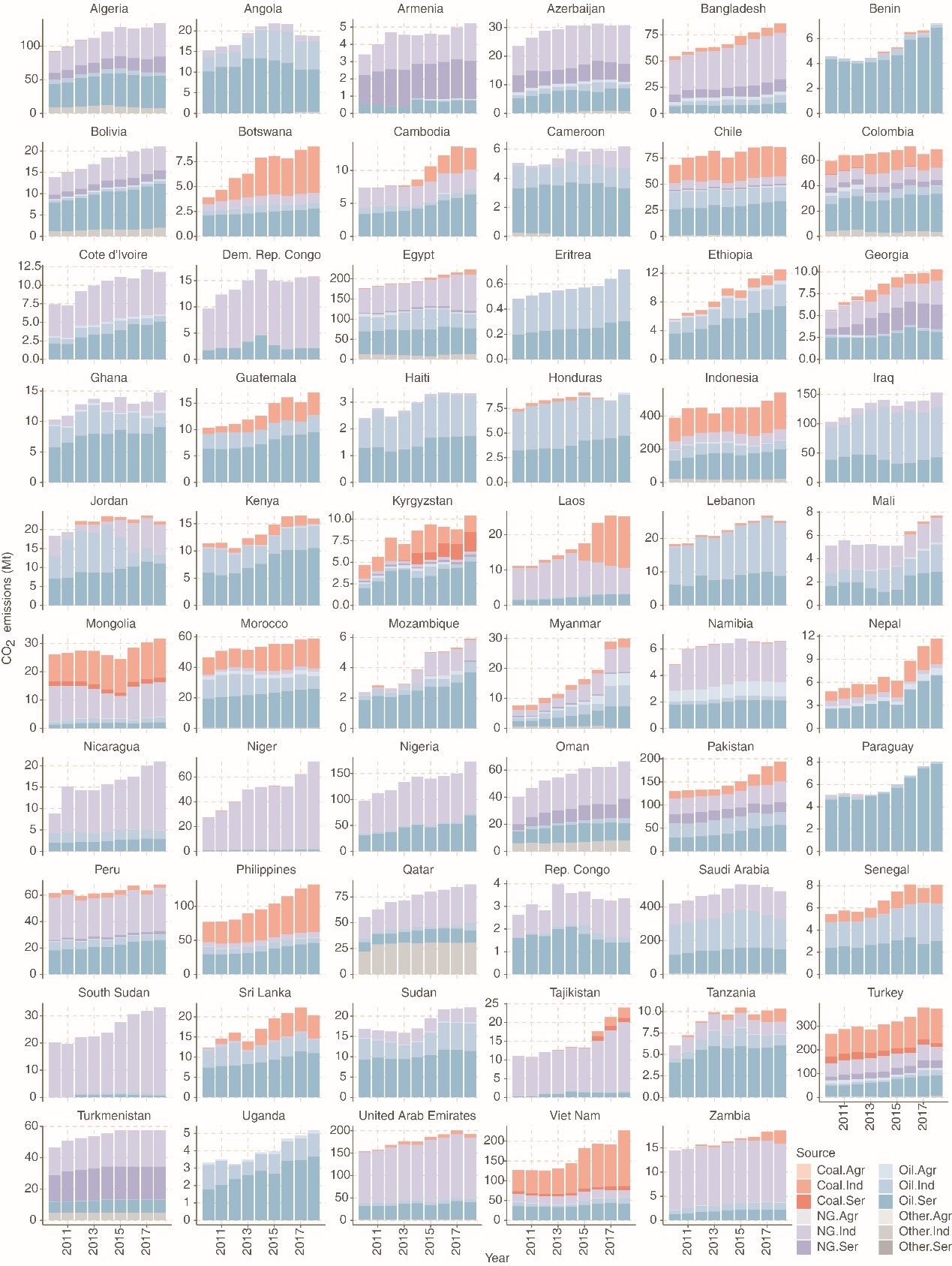


Fig. S1 CO_2_ emissions by fuel type and industry from 2010 to 2018, in million tons. The emissions from coal, oil, natural gas (NG) and other energy types are shown in oranges, blues, purples and greys, respectively; the light to the dark show the emissions from agriculture (Agr), industry (Ind), and services (Ser).

CO_2_ emissions by sector of the emerging emitters

Economic and industry-related drivers contributed largely to the emissions growth of the emerging emitters. The CO_2_ emissions by sector from 2010 to 2018 are shown in Fig. S2 (in percentage). Over 2010-2018, the emerging emitters show great shares of the electricity generation sector (orange color in Fig. S2) and the transportation sector (e.g. dark blue for road transportation in Fig. S2). However, it may be worse for the climate since the ongoing construction of the infrastructure of the emerging emitters would contribute larger emissions in the future, especially power plants and the rising demand of oil-fueled vehicles.


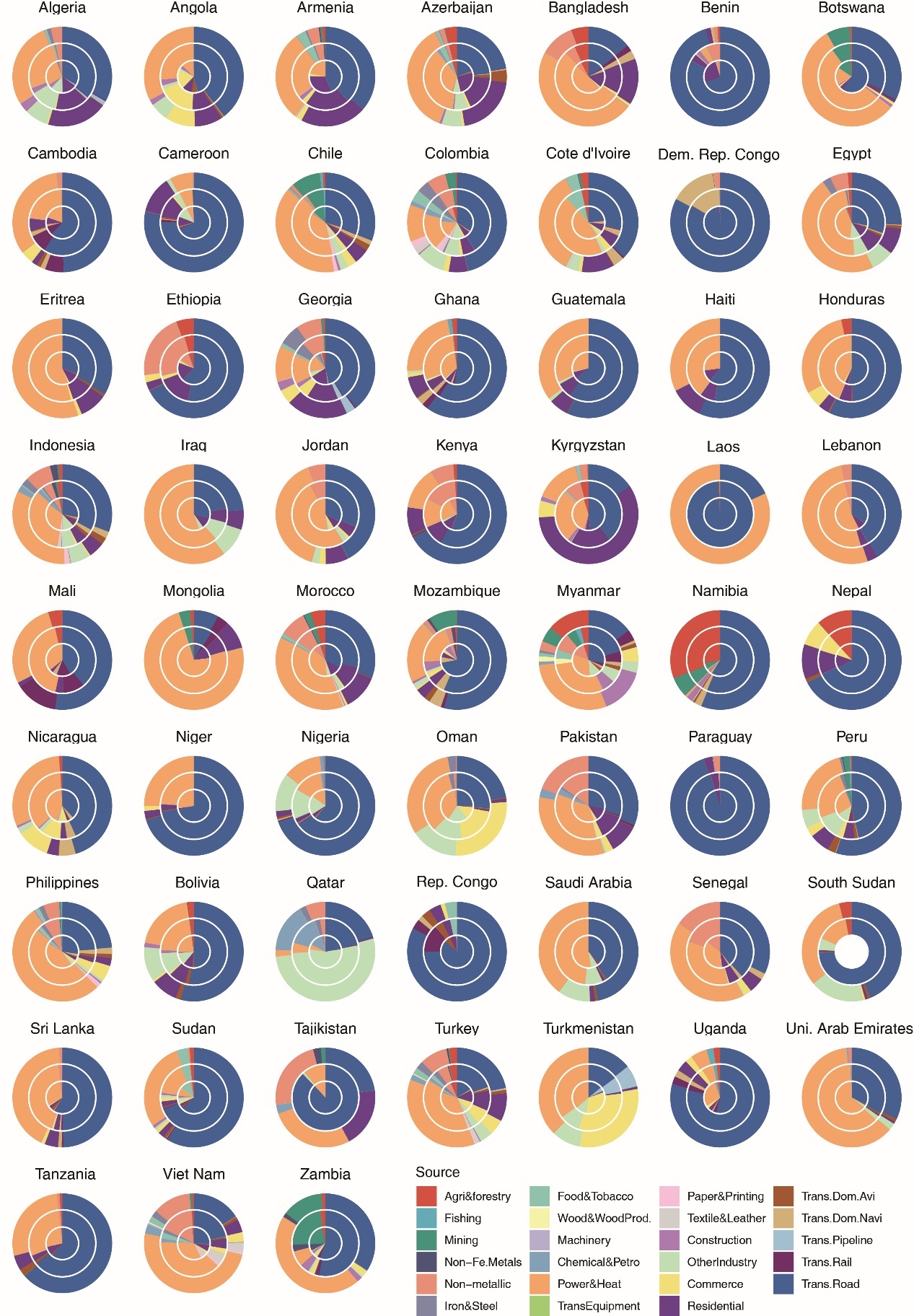


Fig. S2 Shares of CO_2_ emissions by sector from 2010 to 2018, by percentage. The emissions from each sector in 2010, 2014 and 2018 are shown from the inner circle to outer ones.

Drivers of emission surges in the 59 emerging emitters

Here we present the driving forces of emission change in the emerging emitters including the 20 countries already presented in the main text Figure 2, with the contributions of major driving forces to the emission changes (Figs.S3-S6).


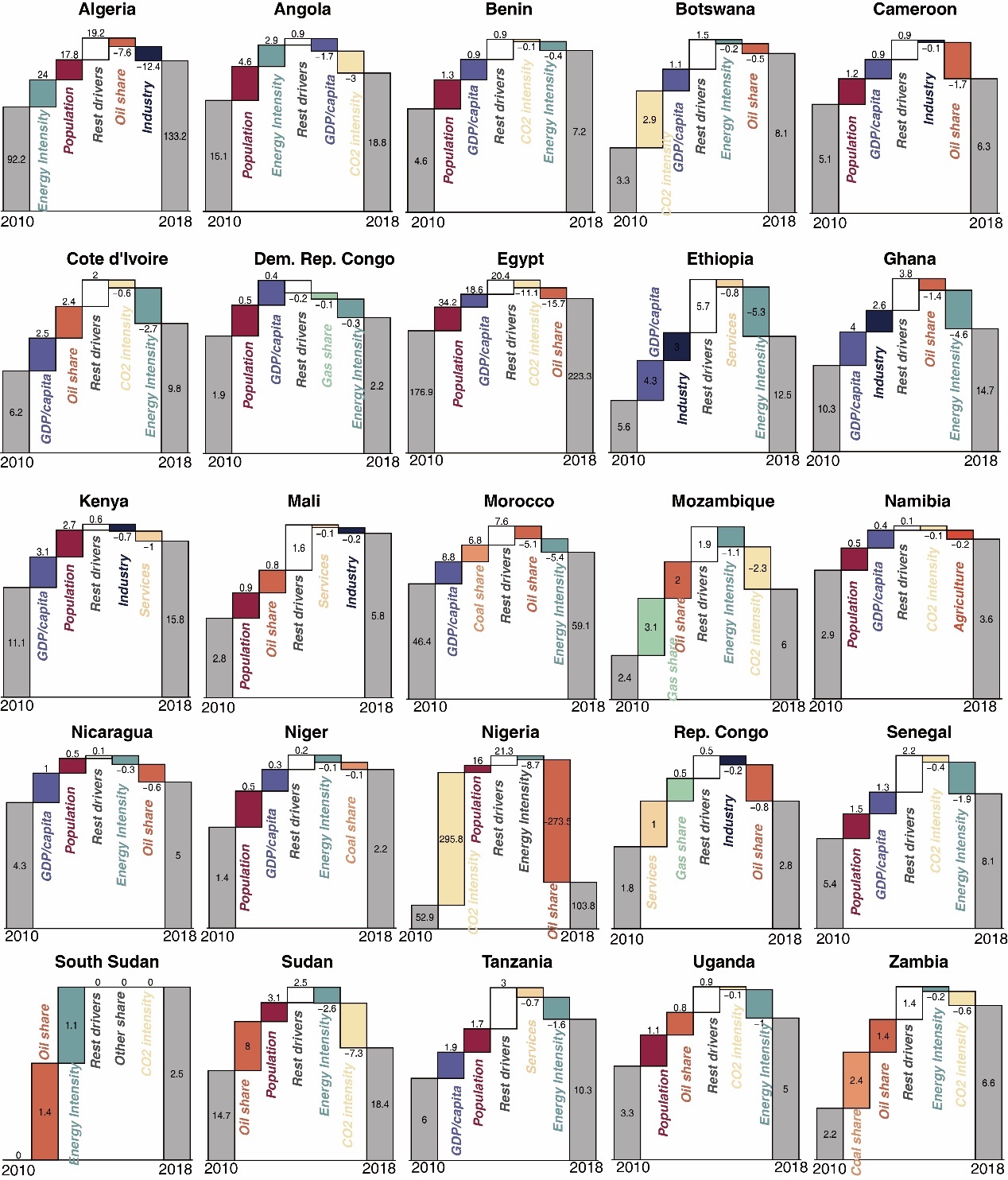


Fig. S3 Driving forces of emission changes of the emerging emitters in Africa. Numbers are the emissions or emission changes from each driver, in Mt. Note: the driving force decomposition of Eritrea is unavailable due to lack of data.


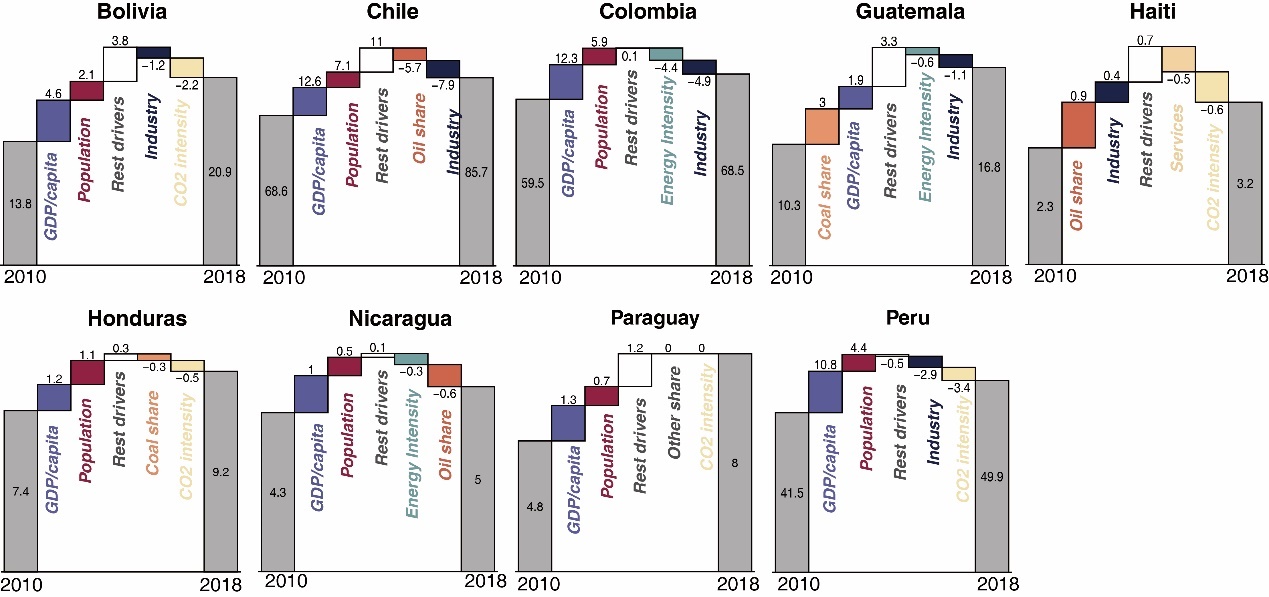


Fig. S4 Driving forces of emission changes of the emerging emitters in Latin America. Numbers are the emissions or emission changes from each driver, in Mt.


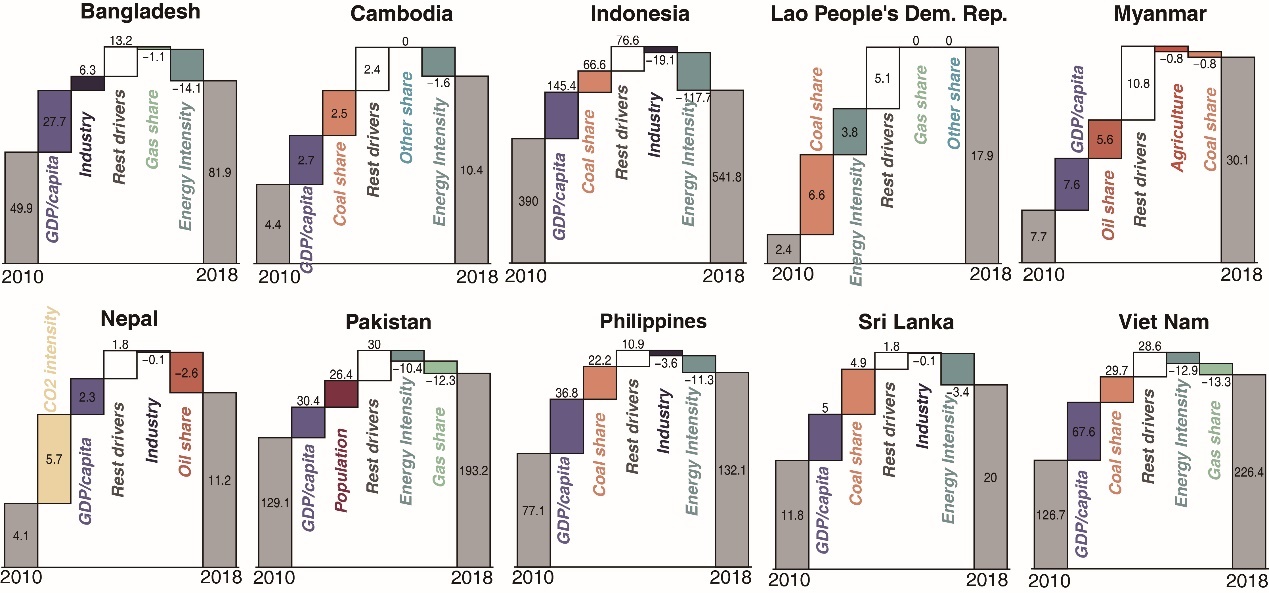


Fig. S5 Driving forces of emission changes of the emerging emitters in South and Southeast Asia. Numbers are the emissions or emission changes from each driver, in Mt.


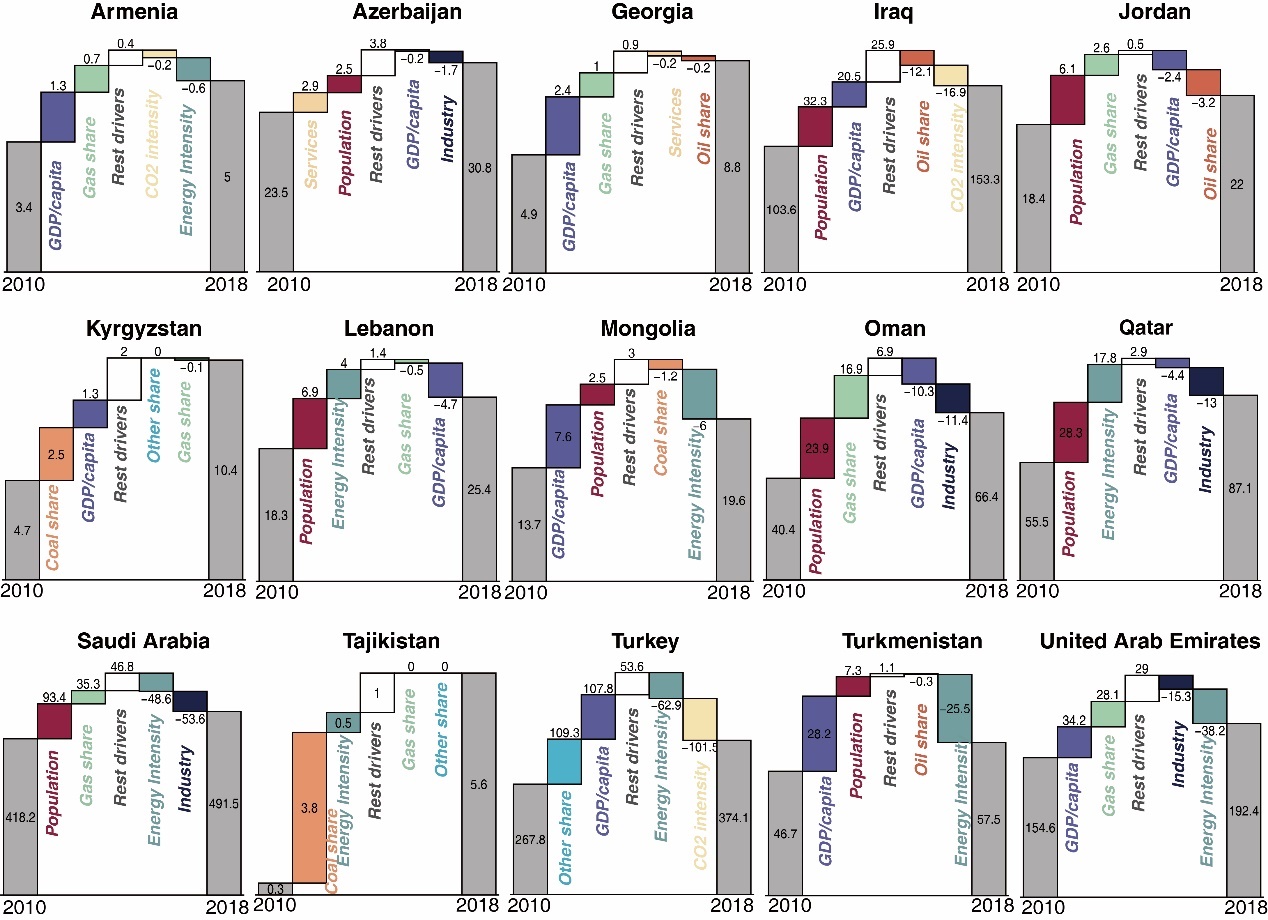


Fig. S6 Driving forces of emission changes of the emerging emitters in Other Asian regions. Numbers are the emissions or emission changes from each driver, in Mt.

Detailed descriptions about the six case countries: Myanmar, Ethiopia, Vietnam, Uganda, Mongolia, and Peru.

**Myanmar** (Figure 2 k): Benefiting from political and economic reform after 2011, Myanmar finally stepped into political stability and was able to focus on domestic economic development: from 2011 to 2018, Myanmar’s GDP keeps an increasing ratio staying around 7% and reached 71.2 billion dollars in 2018. However, along with the economic development, CO_2_ emission was also seen with an increment of up to 300% from 2010 to 2018 level. Therefore, the booming economic development after 2011 has also been seen with an environmental sacrifice to some extent. Comparing with 2010, Myanmar has released 22.5 Mt CO_2_ more in the year of 2018, with per capita GDP, oil consumption, and energy intensity as the top three contributors. Generally, the GDP development is the largest driving force of emission increment, due to the market booming brought by reform. Next, the oil-based emission implies the energy structure of Myanmar become oil-oriented, especially in construction, power generation, manufacturing, and household sectors. Besides, the industry is also of great importance to drive up CO_2_ emission, indicating that Myanmar is in its industrialization and its economic development has been found fundamentally contributed by the manufactory industry. As one of the countries with the fastest-growing CO_2_ emission, Myanmar still has great growth potential in its future emission as well as pressure in climate change mitigation.

CO_2_ emissions and GDP (Fig. S7a)

Benefiting from political and economic reform after 2011, Myanmar stepped into political stability and focused on domestic economic development. From 2011 to 2018, Myanmar keeps an GDP increasing ratio staying around 7%, and domestic industry is being ushered in unprecedented opportunities for development. In the year of 2016, Myanmar has been witnessed with a 90.9% and 74.2% increment on Aircraft, spacecraft, boats and Electrical equip parts, respectively. Here, Myanmar's aviation industry booms largely drive up the corresponding export to Sweden, Singapore, Vietnam, Ireland, etc. However, along with the economic development, CO_2_ emission in Figure*-a was also seen with an increment up to 300% from 2010 to 2018 level. Therefore, the booming economic development after 2011 also been seen with environmental sacrifice to some extent.

Emission drivers (Fig. S7b)

Benefiting from political and economic reform after 2011, Myanmar finally stepped into political stability and was able to focus on domestic economic development: from 2011 to 2018, Myanmar’s GDP keeps an increasing ratio staying around 7% and reached 71.2 billion dollars in 2018. However, along with the economic development, CO_2_ emission was also seen with an increment of up to 300% from 2010 to 2018 level. Therefore, the booming economic development after 2011 has also been seen with an environmental sacrifice to some extent. Comparing with 2010, Myanmar has released 22.5 Mt CO_2_ more in the year of 2018, with per capita GDP, oil consumption, and energy intensity as the top three contributors. Generally, the GDP development is the largest driving force of emission increment, due to the market booming brought by reform. Next, the oil-based emission implies the energy structure of Myanmar become oil-oriented, especially in construction, power generation, manufacturing, and household sectors. Besides, the industry is also of great importance to drive up CO_2_ emission, indicating that Myanmar is in its industrialization and its economic development has been found fundamentally contributed by the manufactory industry. As one of the countries with the fastest-growing CO_2_ emission, Myanmar still has great growth potential in its future emission as well as pressure in climate change mitigation.

Emissions by sector (Fig. S7c)

Fig. S7c gives out sectoral emission ratio by the year of 2010, 2014 and 2018. Interestingly, CO_2_ emission from agricultural has enlarged its ratio from 4.6% (2010) to 13.8% (2018). This part has been found as the comprehensive consequence of opening agriculture market and promoted agriculture mechanization. Since the economic opening of 2011, the main rice growing zones of the Delta and Dry Zone of Myanmar were the first to begin to mechanize, with mechanization subsequently spilling over into areas of the Dry Zone growing non-rice crops, and to upland areas[19]. Numbers of agricultural machinery supply outlets was seen with a rapid growth took place from 2014-2018, during which 61% of all stores were established. Agriculture mechanization was not triggered by direct financial incentive, as well as the consequence of rural-urban migration, which is found up to 80% in 2015 and simultaneously brought labor shortage in rural area. However, the articulation machine promoting such as Two-wheel tractors, Four-wheel tractors and water pump also have stimulated the energy demand to agriculture machine operation especially for oil product, which bring new concern to corresponding CO_2_ emission.

Emissions by fuel type and industry (Fig. S7d)

In Myanmar, the absolute coal-based emission always stays stable although slightly increment can be observed around 2018, while the structural ratio of coal-based emission decreased dramatically from 2010 to 2018. In the contrary, the natural gas and oil-based emission have increased largely. For example, oil-based emission has increased by 47.6%, from the level of 2010 to 2018. In the year of 2018, the oil-based emission was up to 61% of the total 30 Mt-CO_2_. Therefore, it is not hard to define that Myanmar’s economic development is majorly powered by oil consumption, rather than the substantial natural gas produced domestically. From the sectoral perspective, Myanmar’s CO_2_ emission is emitted by second industry mostly, which can be inferred than Myanmar is still lead by manufacturer industry, rather than the service industry.

Trade pattern (Fig. S7e)

In retrospect of selected period, Myanmar’s economic has largely boomed by the reform the benefit a lot from the stable political environment. For example, the opening of demotic agriculture market largely stimulates the import of seed, equipment and fertilizer that contributing to market booming, which contributes the import ratio of Agriculture, forest and fishing industry to roar up to 75% around 2012 than previous year. Myanmar’s major trade pattern has expanded from Asian countries to European counties, such as Sweden and Ireland have built a series of new connections in terms of aircraft and shipping manufactory. Although Myanmar is abundant in natural gas resource, the natural gas product is majorly used for export, rather than domestic consumption. Except traditional agricultural products like rice and beans, the clothing industry also become another important trigger of economic development as well as major export product. To the import, the mechanism and other chemical product are always found as the major products for meeting domestic demand.


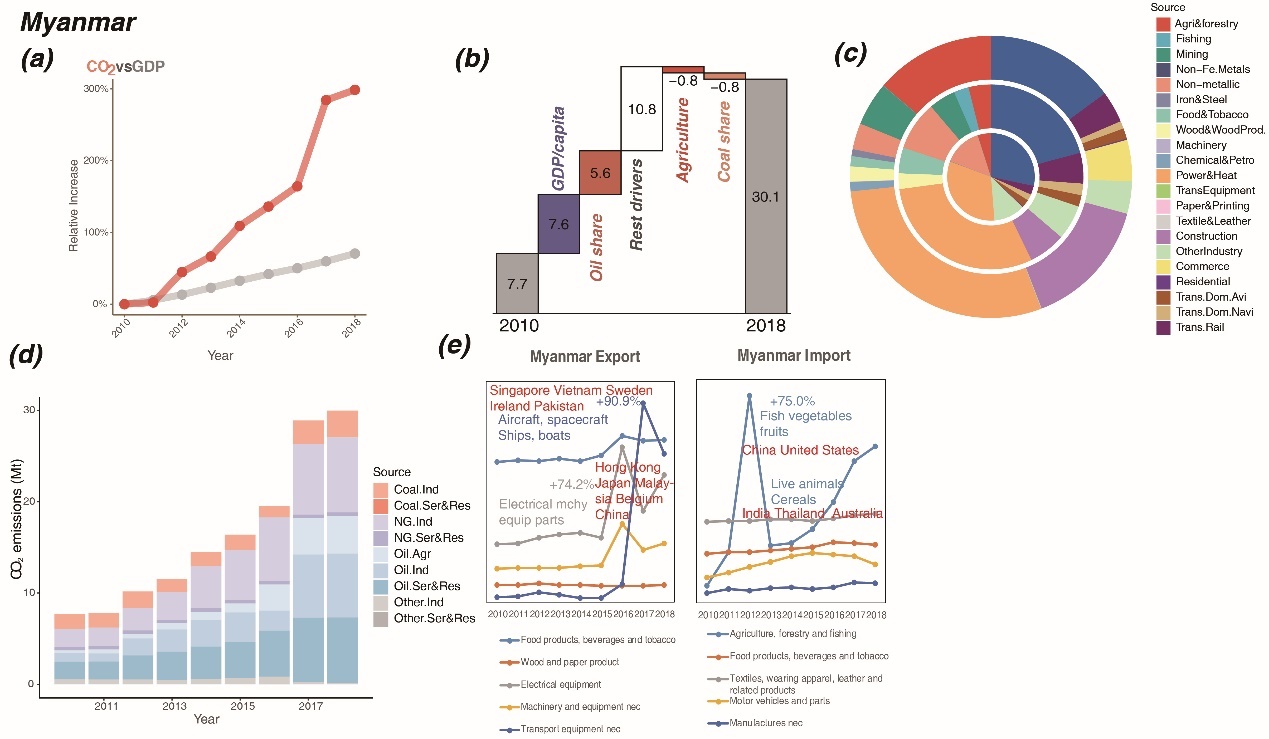


Fig. S7 Relative increase of CO_2_ emissions and GDP (a), emissions change of main drivers (b, in Mt), emissions by sector (c, in 2010, 2014 and 2018 from the inner circle to outer ones), emissions by energy and industry (d), and international trade changes (e) of Myanmar.

**Ethiopia** (Figure 2e): Over 2010-2018, Ethiopia enjoyed an economic boom with an annual growth rate of 10% (84.4 billion dollars in 2018) while at a cost of CO_2_ emissions rising by 11% each year. From 2010, Ethiopia experienced the Growth and Transformation Plan (GTP), which encouraged the large-scale foreign investment in agriculture and industry and stimulated economic growth and industrialization. Therefore, with the extraction of minerals (gold, salt, precious stones, fuels, etc.) and the infrastructure development under the GTP, the booming industry and growing GDP per capita became the largest drivers to the emission growth (summed 7.3 Mt increment). As the road and rail transport and power generation were highlighted in the GTP targets, the expanded use of oil in transport and the higher use of coal in manufacture contributed 3.7 Mt emissions. Population growth also drove the emission growth, as the population growth rate of Ethiopia reached 2.7% per year. The upsurge of population leads to the growing demands for resources, products, and services, and therefore stimulates energy consumption and CO_2_ emissions. Energy intensity decline, as a result of electrification in commerce and household, led to a 5.3 Mt emission reduction over the period.

CO_2_ emissions and GDP (Fig. S8a)

The growth trends of GDP and CO_2_ emissions are shown in Fig. S8a. During the last two decades, Ethiopia has undergone huge structural and economic changes. The share of industry in the gross domestic product increased significantly from 9.44% in 2010 to 27.31% in 2018. With a shift from agriculture to manufacturing in recent years, especially following policies which include the Growth and Transformation Plan (GTP), Ethiopia has experienced high economic growth, averaging 9.8% a year from 2010-2018. Rapid economic growth was also underpinned by high levels of foreign direct investment, which stood at $3.6bn in 2017 compared to $109m a decade earlier. In step with economic development, Ethiopia's CO_2_ emissions have been growing at a slightly faster pace compared to GDP since 2013, with an average growth rate of 10.8%, indicating the process of industrialization and higher emission intensity[20].

Emission drivers (Fig. S8b)

Over 2010-2018, Ethiopia enjoyed an economic boom with an annual growth rate of 10% (84.4 billion dollars in 2018) while at a cost of CO_2_ emissions rising by 11% each year. From 2010, Ethiopia experienced the Growth and Transformation Plan (GTP), which encouraged the large-scale foreign investment in agriculture and industry and stimulated economic growth and industrialization. Therefore, with the extraction of minerals (gold, salt, precious stones, fuels, etc.) and the infrastructure development under the GTP, the booming industry and growing GDP per capita became the largest drivers to the emission growth (7.3 Mt increment). As the road and rail transport and power generation were highlighted in the GTP targets, the expanded use of oil in transport and the higher use of coal in manufacture contributed 3.7 Mt emissions. Population growth also drove the emission growth, as the population growth rate of Ethiopia reached 2.7% per year. The upsurge of population leads to the growing demands for resources, products, and services, and therefore stimulates energy consumption and CO_2_ emissions. Energy intensity decline, as a result of electrification in commerce and household, led to a 5.3 Mt emission reduction over the period.

Emissions by sector (Fig. S8c)

Transportation accounted for the largest share of emissions, about 50 percent, while nonmetal manufacturing has fastest growth trend. According to the GTP, the government had been renewing focus on natural resource and raw material industries such as gold, oil, gas, potash, and gemstones from 2010, resulting in a huge increase in non-metallic. The export of salt, sulphur, earth & stone and plaster increased over 100 times from 984,446$ (in 2010) to 11,283,813$ (in 2018). Improved cook-stoves, universal electrification, and efficient lighting as measures put in place by Ethiopia, have gone a long way to improve household energy efficiency in recent years. Also, extreme drought since 2015 threw millions of people into famine and other crisis, which led to the sharp decrease in residential consumption and emissions. CO_2_ emissions in the power and heat sector have declined as a result of the rapid development of hydropower and the improvement of energy conversion efficiency. Electricity generation by hydro grew from 4931GWh in 2010 to 13018GWh in 2018, and the share of renewables (hydro, wind and solar) in power generation has reached 100% since 2012. Emission shares of agriculture & forestry and commerce remained stable over time.

Emissions by fuel type and industry (Fig. S8d)

Ethiopia’s main energy supply is oil products and coal products, of which oil products are the main source of energy. The main part of Ethiopia's economic plan is to strengthen infrastructure construction. With the rapid development of infrastructure construction in Ethiopia, the industrialization process is constantly advancing, and the demand for cement is increasing. This caused the CO_2_ emissions from non-metallic sector has the fast growth. In addition, because Ethiopia is a land locked country, most of the transportation is completed by rod transportation, which lead road transportation has the largest proportion of CO_2_ emissions.

Trade pattern (Fig. S8e)

Fig. S8d shows the time series of the trade volume of the sectors with the largest change ratio of import and export in Ethiopia from 2010 to 2018. Ethiopia earns most of its foreign exchange from primary industries, such as coffee, oily seeds and manufacturing products, among which rubber and plastic products saw an apparent increase. Exports of transport services have been growing steadily since 2010. The national airline serves destinations in Ethiopia and throughout the African continent, as well as nonstop service to all over the world. We could see a sharp increase in imports of transportation equipment and industrial products, including aircraft or spacecraft from Germany and United States, railway/tramway locomotive from Belgium, as well as other industrial products such as petroleum oils, fertilizers, nuclear reactors and boilers. The import of transportation equipment increased 77.9% from 2010 to 2018 to support its transportation.


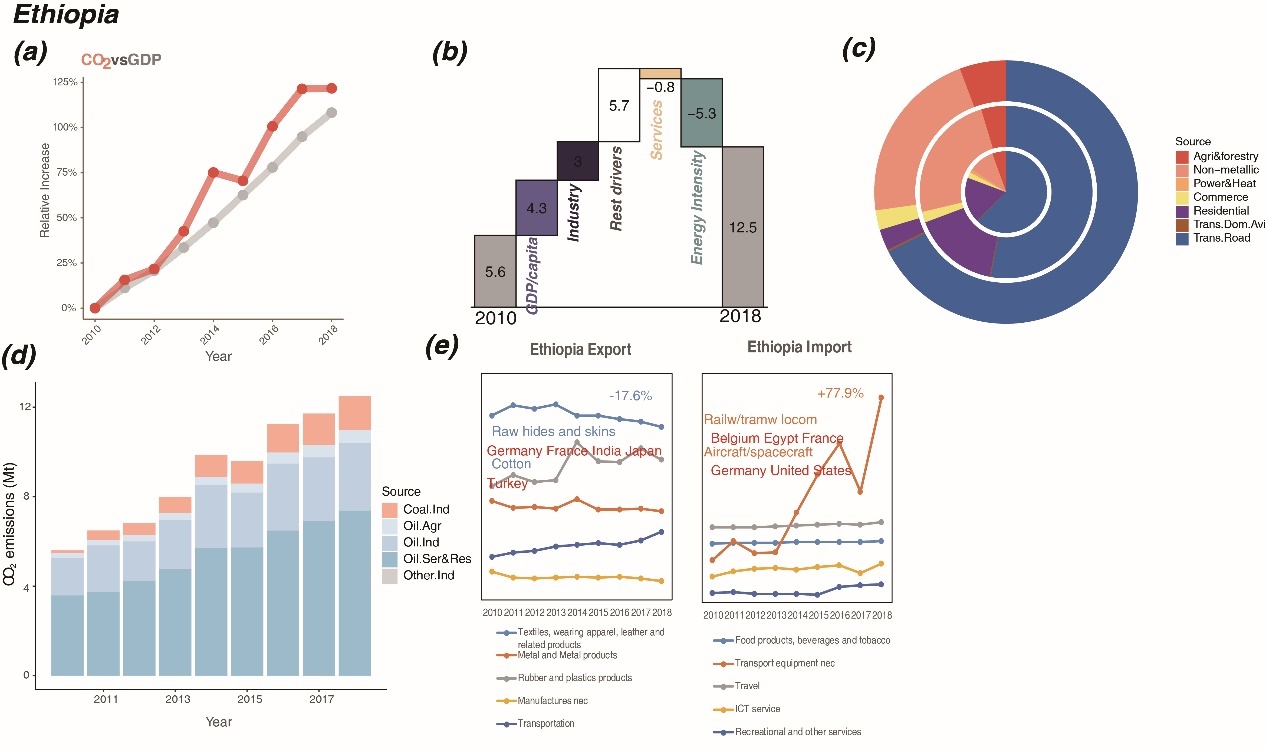


Fig. S8 Relative increase of CO_2_ emissions and GDP (a), emissions change of main drivers (b, in Mt), emissions by sector (c, in 2010, 2014 and 2018 from the inner circle to outer ones), emissions by energy and industry (d), and international trade changes (e) of Ethiopia.

**Vietnam** (Figure 2g): Vietnam experienced rapid growth of both GDP and CO_2_ emission at annual growth rates of 6% and 8%, respectively. In 2018, Vietnam’s GDP reached up to 245.2 billion dollars and CO_2_ emissions reached to 226.7 Mt. During the journey of Vietnam’s emission surging, the increasing GDP per capita drove the most emission growth (67.8 Mt, 68.1% of the total increment). With fast-growing production and exports of textile, real estate, transport, and electrical equipment parts, Vietnam achieved fast and firm economic growth, in which the industry and services contributed 10 Mt emissions increment, and the related energy consumption turned to coal (in power supply) and oil (in textile and electrical parts production) that led to 36.7 Mt incremental emissions. Population growth drove the emission growth by 14 Mt as well, because of the enlarged household demand of the increased population (from 88 million to 97 million, by 1% per year). To sum up, as one of the largest factories for the world with a carbonized energy structure and large population, Vietnam has great potential to continue to surge its CO_2_ emission in the near future.

GDP and CO_2_ emissions (Fig. S9a)

The average annual growth rates of Vietnam's GDP and CO_2_ emissions from 2010-2018 are +6.21% and +7.54% respectively. And more importantly, the growth rate of CO_2_ emissions has begun to increase sharply since 2013, up to 226.5 Mt in 2018. This is mainly due to Vietnam's active trade and opening policy, such as Establishment of ASEAN Free Trade Area(2010), Free trade between Vietnam and South Korea(2015-2016), and carrying out Vietnam Europe free trade negotiations actively. These have attracted a lot of FDI and stimulate the economic growth. Specifically, from 2010 to 2018, the annual compound growth rate of Vietnam's FDI absorption agreement funds will reach 7%, among which, the manufacturing industry's annual compound growth rate of FDI absorption agreement funds is as high as 17%.

Emission drivers (Fig. S9b)

Vietnam experienced rapid growth of both GDP and CO_2_ emission at annual growth rates of 6% and 8%, respectively. In 2018, Vietnam’s GDP reached up to 245.2 billion dollars and CO_2_ emissions reached to 226.7 Mt. During the journey of Vietnam’s emission surging, the increasing GDP per capita drove the most emission growth (67.8 Mt, 68.1% of the total increment). With fast-growing production and exports of textile, real estate, transport, and electrical equipment parts, Vietnam achieved fast and firm economic growth, in which the industry and services contributed 10 Mt emissions increment, and the related energy consumption turned to coal (in power supply) and oil (in textile and electrical parts production) that led to 36.7 Mt incremental emissions. Population growth drove the emission growth by 14 Mt as well, because of the enlarged household demand of the increased population (from 88 million to 97 million, by 1% per year). To sum up, as one of the largest factories for the world with a carbonized energy structure and large population, Vietnam has great potential to continue to surge its CO_2_ emission in the near future.

Emissions by sector (Fig. S9c)

The electricity and heat sectors generate the largest carbon dioxide emissions, and the growth rate of their carbon dioxide emissions is also rapid especially from2016 to 2018. Since 2016, Vietnam's power industry, including renewable energy, has shown signs of stagnation, while Vietnam's economy has maintained rapid growth, and the demand for electricity has also increased. During the period of 2011-2015, the average annual growth rate of Vietnam's electric power investment was 10%, among which the most is in thermal power generation. In 2018, Vietnam's coal-fired thermal power generation accounted for 32.6% of total power generation, which is the main factor of increasing coal consumption.

Emissions by fuel type and industry (Fig. S9d)

Coal is the main energy source of Vietnam and its proportion is still increasing during this research period. In the use of coal, the sectors that use coal the most in Vietnam are power plants, non-metal manufacturing and electrical supply sectors (Fig. S9b). Generally, the usages of coal have increased by 53.5% from 2010 (23.4Mt) to 2018(35.9Mt). However, during this period, there have a fluctuation. Since 2013, the use of coal in these sectors has risen. However, since 2016, there has been a gradual flattening or even a downward trend. This mainly because the supply of domestic coal has fallen (Vietnam’s domestic coal production declined 3.1% in 2016 compared to 2015), and the amount of imported coal has not met the gap. At the same time, it is worth noting that the growth output of the textile and apparel industry, so-called high load energy industry, lead the fossil fuel consumption rise. CO_2_ emissions from the combustion of petroleum products are slightly increased from 2010 to 2018.Transportation consumes the largest proportion of oil. This can be ascribed to the policies of vigorously develop the domestic transportation industry. From 2010-2018, the oil used in transportation has growth from 11.2Mt in 2010 to 11.8Mt in 2018, by rate of 5.06%.

Trade pattern (Fig. S9e)

From the perspective of Vietnam's import side, Vietnam imported a large number of transportation equipment such as railways, trams and passenger cars from 2010-2018, from 24.7 million dollars in 2010 to 49.1 million dollars in 2018 (especially after 2013, mainly due to Vietnam's "North-South High-Speed Railway Program") .At the same time, in addition to continuing to increase exports from the traditional textile and apparel industry during 2010-2018, Vietnam's exports of electrical equipment components are also growing rapidly.


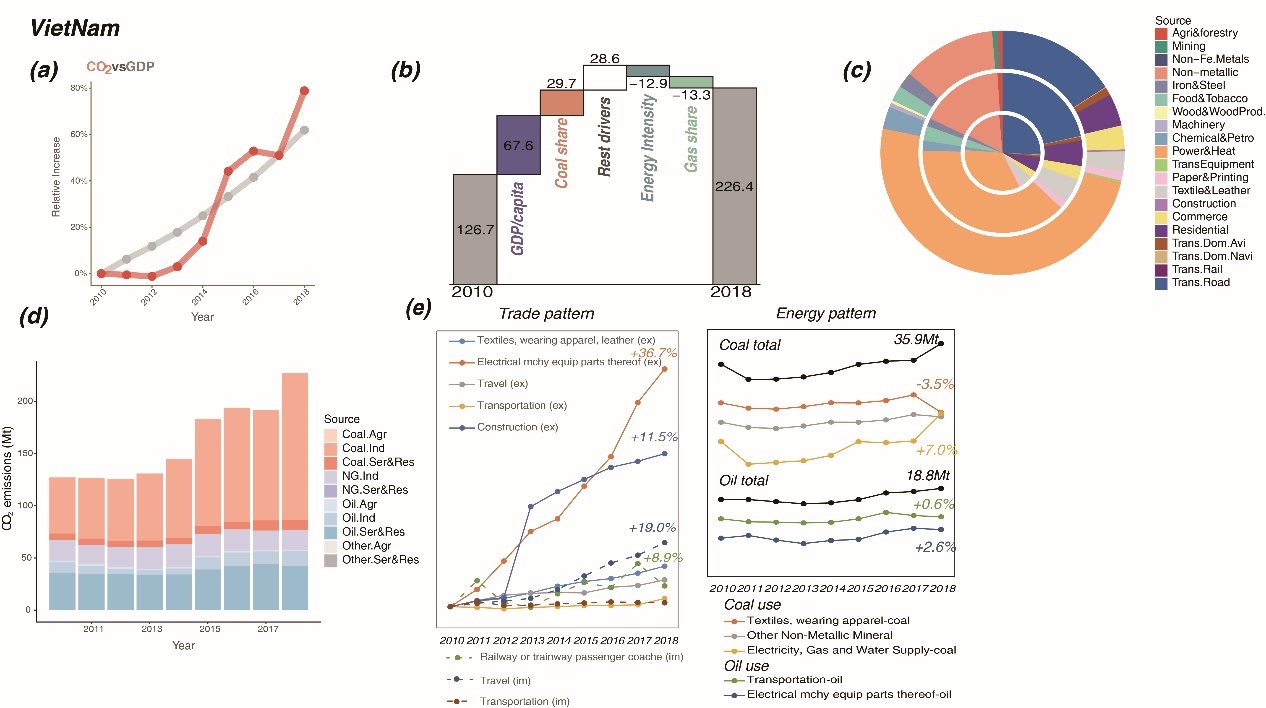


Fig. S9 Relative increase of CO_2_ emissions and GDP (a), emissions change of main drivers (b, in Mt), emissions by sector (c, in 2010, 2014 and 2018 from the inner circle to outer ones), emissions by energy and industry (d), and international trade changes (e) of Vietnam.

**Uganda** (Figure 2 a): Uganda maintained a 5% annual average growth rate in GDP in 2010-2018 under its Five-Year Plans. In 2018, its GDP reached up to 27.5 billion dollars, increasing by around 1.85 times than that in 2010. At the same time, with the boom of domestic economic activities, the carbon emissions also gradually increase year by year with an annual average growth rate of 6%, increasing to 5 Mt by 1.45 times than 2010. Increased population and oil consumption are the main impetus driving the growth of emissions in Uganda, which contributes 37.0% (1.1Mt) and 27.6% (0.8Mt) to CO_2_ emission increment, respectively. Uganda maintains a 3.6% annual average population growth rate over 2010-2018, and its net population growth totals 10.29 million. Over the period, oil consumption increased with an annual growth rate of 7.1%, which gives rise to the increase of CO_2_ emissions as well. Apart from that, the economic expansion effect and the development of services also make for the growth of CO_2_ emissions during the research period. The output of the services increased by 32 times over 2010-2018, mainly driven by the rising transport sector. Investments in renewable energy benefited Uganda in lower energy intensity and that led to 1 Mt less emission over the period.

GDP and CO_2_ emissions (Fig. S10a)

Uganda maintained 5.1% annual average growth rate in GDP in 2010-2018. In 2018, its GDP reached up to 122694 billion shillings at 2016 constant price, increasing by around 1.46 times than that in 2010. It is primarily ascribed to overseas financial aid to Uganda and also the industrial policy of the Uganda government. As of the 21st century, the Uganda government takes active measures to execute the policies promulgated by IMF and the World Bank, which greatly attracts the financial aid from more foreign countries. In addition, Uganda begins to implement its new five-year national plan since 2010, and successfully schemes the new national development plan in 2015. By emphasizing the development of agriculture, energy, service industry and strategic infrastructure, the plan is aimed at advancing the economic growth of the country, in particular the service industry. At the same time, with the boom of domestic economic activities, the carbon emissions also gradually increase year by year with an annual average growth rate of 5.9%, increasingly to 4.8 Mt by 1.45 times than 2010.

Emission drivers (Fig. S10b)

Uganda maintained a 5% annual average growth rate in GDP in 2010-2018 under its Five-Year Plans. In 2018, its GDP reached up to 27.5 billion dollars, increasing by around 1.85 times than that in 2010. At the same time, with the boom of domestic economic activities, the carbon emissions also gradually increase year by year with an annual average growth rate of 6%, increasing to 5 Mt by 1.45 times than 2010. Increased population and oil consumption are the main impetus driving the growth of emissions in Uganda, which contributes 37.0% (1.1Mt) and 27.6% (0.8Mt) to CO_2_ emission increment, respectively. Uganda maintains a 3.6% annual average population growth rate over 2010-2018, and its net population growth totals 10.29 million. Over the period, oil consumption increased with an annual growth rate of 7.1%, which gives rise to the increase of CO_2_ emissions as well. Apart from that, the economic expansion effect and the development of services also make for the growth of CO_2_ emissions during the research period. The output of the services increased by 32 times over 2010-2018, mainly driven by the rising transport sector. Investments in renewable energy benefited Uganda in lower energy intensity and that led to 1 Mt less emission over the period.

Emissions by sector (Fig. S10c)

According to the circular chart, it can be seen that the major emission sector in Uganda is the transportation sector especially the rood transportation. This is because road transportation carries about 95% of the country's cargo transportation and about 99% of passenger transportation[21]. What’s more, CO_2_ emissions of the road transportation and its share are increasing from 2010-2018. This is due to the Uganda Road Sector Support Initiative (URSSI) implemented in 2010, the investment of road transportation increased from 2000 million dollars in 2011 to about 9500 million dollars in 2017 with 21.5% average growth rate[22]. In addition, the proportion of CO_2_ emissions from the power and heating industry has shown an overall trend of increasing first and then decreasing during the period 2010 to 2018.This mainly because the Uganda electricity system partly relied on the hydrogen power, which is easily affected by climate change. During drought period (2011-2012), due to the drop in the water level of Lake Victoria, the hydroelectric power plant can only use about half of the installed capacity, resulting in inadequate domestic electricity supply. Uganda increase thermal power generation about 150MW in order to fill the power demand gap, which also increased the CO_2_ emissions in electricity heat sector. However, after the successful construction of the Bujagali 250MW hydropower plant in 2012[23] and the water level of Lake Victoria is in a stable state. The Ugandan government gradually reduced the amount of electricity generated by thermal power plants and then hydropower accounts for about 75.6% of the total installed capacity of 983MW in 2018[23]. And therefore, only when hydroelectric power generation is insufficient to meet the electricity demand during periods of drought, the operation of thermal power generation will be increased in order to maintain power grid steady. As a result, the CO_2_ emissions generated by the power sector in Uganda show to increase first and then decrease.

Emissions by fuel type and industry (Fig. S10d)

As can be seen from the Emissions by fuel type and industry, oil is the only energy consumption category in Uganda. Between 2010 and 2018, CO_2_ emissions from oil consumption increased in fluctuations. In 2011-2012, there was a very significant decline of CO_2_ emissions. This is because a severe drought in Uganda in 2011. The drop in water levels led to a shortage of hydroelectric power, which is unable to meet the supply of industrial electricity. In other words, industrial production was affected to a certain extent, which inevitably led to a decline of CO_2_ emissions from the second industry. Another aspect, it had a major impact on agricultural sector production, resulting in a significant decline in the harvest of agricultural products that year. As the climate stabilized after 2012, industrial production gradually returned to steady growth. More importantly, the CO_2_ emissions emitted by service and residential sector accounts for more than half of the total and increase steadily in Uganda during the research period. This is mainly due to population and service output increase. Uganda maintains 3.6% annual average population growth rate in 2010-2018, and its net population growth totals 10.29 million[24]. The upsurge of population leads to the growing demands for resources, products and services, and therefore stimulates energy consumption and CO_2_ emissions. On another side, that is benefited by Uganda’s stable political environment and its development policies for the service industry in 2010-2018, during which the output value of the service industry increased from 32225 billion to 52946 billion shillings at 2016 constant price, and corresponding proportion in GDP also reached 43.15% in 2018. And therefore, the demand for fuel brought by population and the government's strong industrial policies have led to a steady increase in CO_2_ emissions from the service and residential sector during this period.

Trade pattern (Fig. S10e)

In terms of foreign trade, Uganda’s main exports goods are gold and related products (39.6%), followed by coffee and other economic crops (11.7%), and the main exporting countries are the United Arab Emirates and Kenya[25]. Since 2016, gold exports have rocketed in the Uganda exceeding the coffee became the largest export goods, and gold worth 1.68 billion dollars was shipped out of Uganda in 2018– over 60 times what the country was exporting a decade. This is because that the Uganda cancel the gold export tax in 2014 and African Gold Refinery (AGR) was built and operated in 2015[26], a first gold production and refiner in east Africa, which is also the main reason for Uganda’s gold production surge. Additionally, Uganda is located in the most fertile land in Africa and close to Lake Victoria, which provides favorable growth conditions for economic crops such as coffee and cotton. Uganda has a large agricultural population, up to 74%, which provides sufficient cheap labor for Uganda’s agricultural production. These have made Uganda's agricultural products of high quality and low price, attracting overseas customers. Furthermore, during this period the manufacturing for aviation and components export has the fastest growth especially after 2016. This is principally because Uganda and Russia jointly established Pro Heli International Service Limited in 2016, a helicopter and maintenance parts manufacturing company, mainly for Russian armed helicopter parts. In terms of import goods[27], 2018 UN customs data showed that Uganda mainly imported petroleum products from the UAE and Saudi Arabia, accounting 17.4% of the total, and imported packaged medicines from India (3.93%)^5^. As for importing countries, Uganda’s largest importing country is China, with a total import value of over US$1.15 billion, including broadcasting equipment (18%) and mobile phones (4.98%). To sum up, Uganda’s main export and import goods are gold and oil respectively. The largest exporter is the UAE, and importer is China.


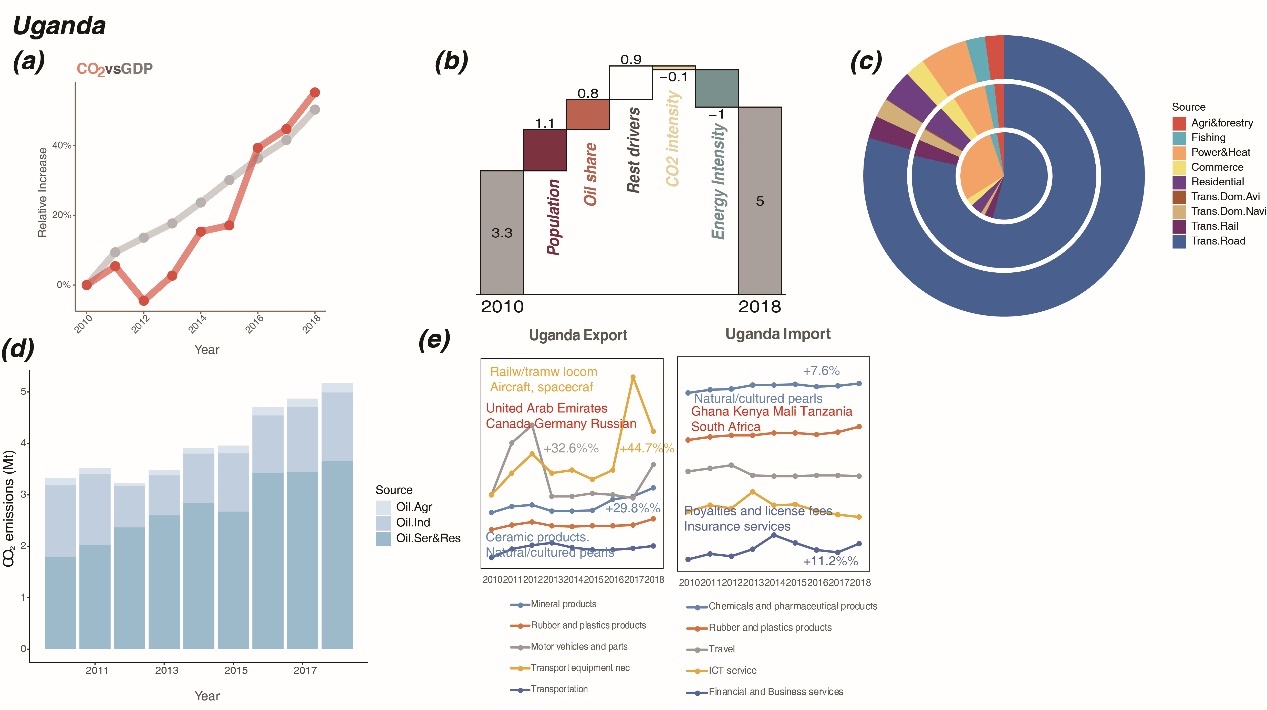


Fig. S10 Relative increase of CO_2_ emissions and GDP (a), emissions change of main drivers (b, in Mt), emissions by sector (c, in 2010, 2014 and 2018 from the inner circle to outer ones), emissions by energy and industry (d), and international trade changes (e) of Uganda.

**Mongolia** (Figure 2 c): Over 2010-2018, under a stable domestic political situation after the 2009 presidential election, Mongolia experienced rapid economic growth with an average annual growth rate of 8%, and its GDP reached 13.1 billion dollars mainly driven by exports (metals and fuels to China). Meanwhile, CO_2_ emissions of Mongolia underwent a rising trend with a cliff-like decline (due to unstable domestic policies, investment, and low price of bulk products in the international market), which showed an average annual growth rate of 5%. GDP per capita, population, and industry are the main drivers of Mongolia’s emission growth. As the pillar industry of the national economy, the mining industry is the essential engine for economic growth: in 2014, mining output value accounted for about 30% of Mongolia’s GDP; the development of such a pillar industry is also crucial for the CO_2_ emission growth. As of 2018, Mongolia’s coal production reached a record high of over 50 million tons, and coal export also reached a record high of 36.3 million tons. That also contributed to the incremental emission of 4.3 Mt driven by industry growth. Mongolia’s population is a major cause of carbon emissions for household consumption due to the poor natural conditions, the cold climate, and the traditional means of heating. It is worth noting that the contribution of energy intensity to carbon emissions has decreased significantly(-11Mt) due to the energy efficiency improvement project of Ulan Bator cogeneration units.

GDP and CO_2_ emissions (Fig. S11a)

According to the results of study period, the CO_2_ emissions of Mongolia shown in Fig. S11a from 2010 to 2018 are basically consistent with the GDP growth level, showing a high-speed upward trend in general, and the GDP growth rate is significantly higher than carbon dioxide emissions. To be more specific, GDP in 2010 was 7.19 bills of US dollars, and in 2018 it was 13.11 bills of US dollars[28], with an average annual growth rate of 7.96%. The rapid growth of Mongolia is due to the fact that after the 2009 presidential election, the domestic political situation is basically stable, and the authorities have insisted on taking the road of stable development to provide a political foundation for economic prosperity. In addition, with the gradual weakening of the impact of the global financial crisis, the global mining industry out of the trough, and the rising price of mineral products in the international market, Mongolia’s economy has recovered rapidly. Moreover, with the implementation of the strategy of “invigorating the country through mining”, the national economy has developed rapidly under the guidance of mining development, meanwhile, carbon emissions increased. However, due to the single industrial structure of Mongolia, once affected by the economic cycle and changes in the international market, the economic situation is relatively unstable. During 2013-2015, affected by internal factors such as unstable domestic policies and investment environment, as well as external factors such as the continuous low price of bulk products in the international market, the economic situation deteriorate rapidly, as a result, the growth rate of the national economy slowed down significantly. At the same time, the carbon dioxide emissions showed a cliff-like decline, but it began to recover slowly in 2016.

Emission drivers (Fig. S11b)

Over 2010-2018, under a stable domestic political situation after the 2009 presidential election, Mongolia experienced rapid economic growth with an average annual growth rate of 8%, and its GDP reached 13.1 billion dollars mainly driven by exports (metals and fuels to China). Meanwhile, CO_2_ emissions of Mongolia underwent a rising trend with a cliff-like decline (due to unstable domestic policies, investment, and low price of bulk products in the international market), which showed an average annual growth rate of 5%. GDP per capita, population, and industry are the main drivers of Mongolia’s emission growth. As the pillar industry of the national economy, the mining industry is the essential engine for economic growth: in 2014, mining output value accounted for about 30% of Mongolia’s GDP; the development of such a pillar industry is also crucial for the CO_2_ emission growth. As of 2018, Mongolia’s coal production reached a record high of over 50 million tons, and coal export also reached a record high of 36.3 million tons. That also contributed to the incremental emission of 4.3 Mt driven by industry growth. Mongolia’s population is a major cause of carbon emissions for household consumption due to the poor natural conditions, the cold climate, and the traditional means of heating. It is worth noting that the contribution of energy intensity to carbon emissions has decreased significantly(-11Mt) due to the energy efficiency improvement project of Ulan Bator cogeneration units.

Emissions by sector (Fig. S11c)

According to the Emission by sector, the main sectors emitted CO_2_ are the power and heat industry, households and transportation. Their emission proportions have not changed significantly for many years (2010, 2014, 2018) accounting for about 72%, 8%, and 7% respectively and emissions from these sectors increased steadily. The main reason is that economic structure and energy structure used by sectors in Mongolia have no changes during this period. Additionally, the CO_2_ emissions from the power and heat industry is still growing the most. Because the Mongolian government has committed to accelerating the construction speed of national power infrastructure, by putting forward a series of plans of reconstruction or expansion of existing power and construction of new power, in order to solve the low efficiency of power sector facilities and shortage of power supply, which restricts economic development. More specifically from 2012 to 2018, Mongolia's installed power generation capacity increased by 30%, among which thermal power installed capacity increased by 40% and the number of power users increased by 4%. In 2018, the installed electric power capacity of Mongolia was 1.1 million kilowatts, of which 93% was thermal power generation, and 5.8% relied on imports. The growth of CO_2_ emissions generated by the transportation sector is the second only to the electricity and heating sector, with the largest increase from railway sector. In 2010, the Mongolian parliament passed the national railway transportation construction plan, which proposed to build 5683.5 km new railway infrastructure in stages[29]. After completion, the railway transportation capacity in Mongolia will be greatly improved, and the railway direct transportation from, Naryn Sukhait and Tavan Tolgoi coal mine, Oyu tolgoi copper and gold mine, and other large mining areas in southern Gobi province of Mongolia to Mongolia border port will be realized.

Emissions by fuel type and industry (Fig. S11d)

Fig. S11c shows that the CO_2_ emissions of Mongolia mainly comes from coal industry and natural gas industry. This is due to the fact that the reserves of coal and oil shale in the country are in the forefront of the world, and coal combustion is the main way to provide electricity and heat energy for local residents. At the same time, in 2013, the Mongolian government proposed to build "five channels" connecting China, Mongolia and Russia, such as railways, highways, oil, electricity and natural gas, and expand trans Mongolian railways and natural gas pipelines to form a comprehensive emission. However, due to the coal industry is the pillar industry of Mongolia, coupled with the lack of domestic funds, the construction of energy transportation system and renewable energy development in Mongolia is slow

Trade pattern (Fig. S11e)

In terms of foreign trade, Mongolia's exports are mainly livestock products and mineral products[30], and mineral products accounted for more than 70 percent of the total exports of Mongolia. This is because Mongolia's economy is mainly dependent on animal husbandry and mining. Additionally, Mongolia's industry is backward and most of its industrial products need to be imported, in order to meet the needs of social development and daily consumption of residents. And therefore, imports include a wide range of consumer and industrial products, such as transport, construction machinery, electrical and electronic products, refined oil, grain, daily necessities, etc. More specially, from 2013 to 2018, the import and export of financial and commercial services grew rapidly. This is due to the increasingly improved domestic investment environment and the in-depth advancement of the "Belt and Road" initiative, which promoted the deepening of regional financial and monetary cooperation. At the same time, imports of vehicles (such as cars and trains, etc.) have also risen significantly. The reason is that the population growth of Ulaanbaatar and other major cities has led to a substantial increase in their car imports, while the large increase in freight vehicles is mainly due to the recovery of mining development. Mongolia has carried out bilateral trade with many countries or regions around the world, and its main exporters are China, Russia, Ukraine, the United States, Canada and other countries. China is the largest trading partner of Mongolia, with a trade volume of nearly 8 billion of US dollars in 2018.


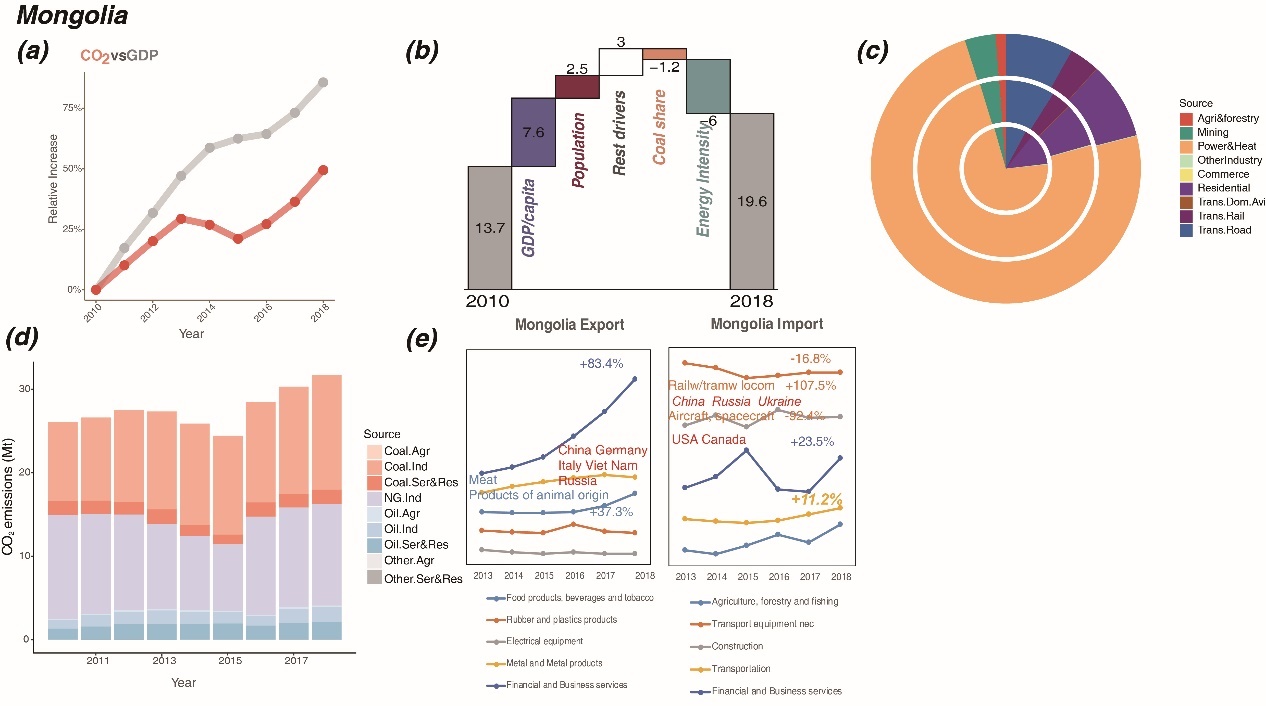


Fig. S11 Relative increase of CO_2_ emissions and GDP (a), emissions change of main drivers (b, in Mt), emissions by sector (c, in 2010, 2014 and 2018 from the inner circle to outer ones), emissions by energy and industry (d), and international trade changes (e) of Mongolia.

**Peru** (Figure 2 b): From 2010 to 2018, Peru's GDP grew at a speed of 4.3% per year, nearly twice as the carbon emissions growth (2.4%). GDP per capita and population growth are the two main factors driving Peru's CO_2_ emission growth. The GDP increased to 222 billion dollars in 2018, resulting from the national infrastructure construction such as transportation highlighted by the Peruvian government: in the past ten years, the Peruvian government has implemented a series of supportive policies including investment, funding, and infrastructure construction to stimulate economic growth which directly improve national economic growth. Meanwhile, with the fast increase of population growth rate from 2010 to 2018 (0.7% in 2010, to 1.5% in 2018), the population growth is undoubtedly the second biggest contributor to Peru's domestic carbon emissions. The absolute increase in the Peruvian population, in part, is due to the young population structure, while another considerable reason is immigration growth. More and more migrants from other countries come to Peru, especially from Venezuela. By 2018, more than three million immigrants have been officially accepted by the Peruvian government. Foreign immigrants will become the future of energy consumption and carbon emissions contributor in Peru. Besides, the expanded transport, household, and commerce sectors contributed to the incremental emission from services, and also pulled the oil consumption, thus the services and the oil use drove a 3.5 Mt CO_2_ emission increment.

GDP and CO_2_ emissions (Fig. S12a)

From 2010 to 2018, Peru's GDP grew more than 40%, nearly twice as the carbon emissions growth (20%). There was a significant fall in CO_2_ emissions in 2017, followed by a slight rise in 2018, but overall growth was much slower than GDP, so Peru was in the sustainable growth category over the past decade.

Emission drivers (Fig. S12b)

From 2010 to 2018, Peru's GDP grew at a speed of 4.3% per year, nearly twice as the carbon emissions growth (2.4%). GDP per capita and population growth are the two main factors driving Peru's CO_2_ emission growth. The GDP increased to 222 billion dollars in 2018, resulting from the national infrastructure construction such as transportation highlighted by the Peruvian government: in the past ten years, the Peruvian government has implemented a series of supportive policies including investment, funding, and infrastructure construction to stimulate economic growth which directly improve national economic growth. Meanwhile, with the fast increase of population growth rate from 2010 to 2018 (0.7% in 2010, to 1.5% in 2018), the population growth is undoubtedly the second biggest contributor to Peru's domestic carbon emissions. The absolute increase in the Peruvian population, in part, is due to the young population structure, while another considerable reason is immigration growth. More and more migrants from other countries come to Peru, especially from Venezuela. By 2018, more than three million immigrants have been officially accepted by the Peruvian government. Foreign immigrants will become the future of energy consumption and carbon emissions contributor in Peru. Besides, the expanded transport, household, and commerce sectors contributed to the incremental emission from services, and also pulled the oil consumption, thus the services and the oil use drove a 3.5 Mt CO_2_ emission increment.

Emissions by sector (Fig. S12c)

The road transport sector is Peru's main emitter, and its emissions are growing fast, especially from 2014 to 2018.It is due to the low turnover rate of the fleet, poor driving habits, insufficient traffic regulations and lack of vehicle maintenance. Additionally, a very bad fuel price policy that does not promote the use of clean fuels. Specially, Peru uses stabilization fund to ease price of fossil fuel volatility. Additionally, CO_2_ emissions from shipping and air transport have also increased. Peruvian government is constantly promoting the renovation and expansion of the coastal railway. On shipping transportation, government also works on a port expansion to meet the needs of commercial shipping. For example, the expansion of Port of Callao and the new construction of Port of Paita. Overall CO_2_ emissions in the transportation sector increased sharply from 2010 to 2018.

Meanwhile, with the fast increase of population growth rate from 2010 to 2018 (0.7% in 2010, to 1.5% in 2018), makes residential energy consumption the second increase to CO_2_ emissions. The absolute increase in the Peruvian population, in part, is due to the young population structure, a large proportion of population are of childbearing age. Another reason is immigration. More and more migrants come to Peru, especially from Venezuela. By 2018, more than three million immigrants have been officially accepted by the Peruvian government. Among them, there are 800,000 international migrants from Venezuela and this number is still growing. Foreign immigrants become the energy consumption and carbon emissions contributor of Peru from 2014 to 2018.

Emissions by fuel type and industry (Fig. S12d)

On the whole, Peru's energy structure has remained stable during this research period. The main energy sources of Peru are natural gas and oil. Peru's oil production is not enough to meet domestic demand. Due to its limited refining capacity, Peru exports crude oil and imports refined oil. Natural gas is mainly used for power generation, automobile fuel and household fuel. In recent years, Peru has been increasing its efforts to explore and develop oil and gas resources, and has signed sixty-eight oil and gas exploration and development contracts in Peru's coastal, mountainous and rainforest areas. The main oil field is Tarala oil field along the northern Pacific coast, and the largest gas field is Camisea gas field in the southeast. In November 2014, PetroChina completed the delivery of major oil and gas assets of Petrobras in Peru, becoming the largest oil company in Peru, accounting for more than half of oil production.

Trade pattern (Fig. S12e)

In terms of foreign trade, Peru had a significant increase in the import of transportation equipment such as railway equipment from China, Spain, Italy, Mexico, and Brazil. The wide application of these vehicles has led to the fast growth of Peru’s transport emission. Great importance the Peruvian government lays on national transportation infrastructure construction. For export, ships and boats are the main export vehicle commodities, especially during 2010-2014. Additionally, the export of motor vehicles has the largest growing, increased by 22.1% from 2010 to 2018.


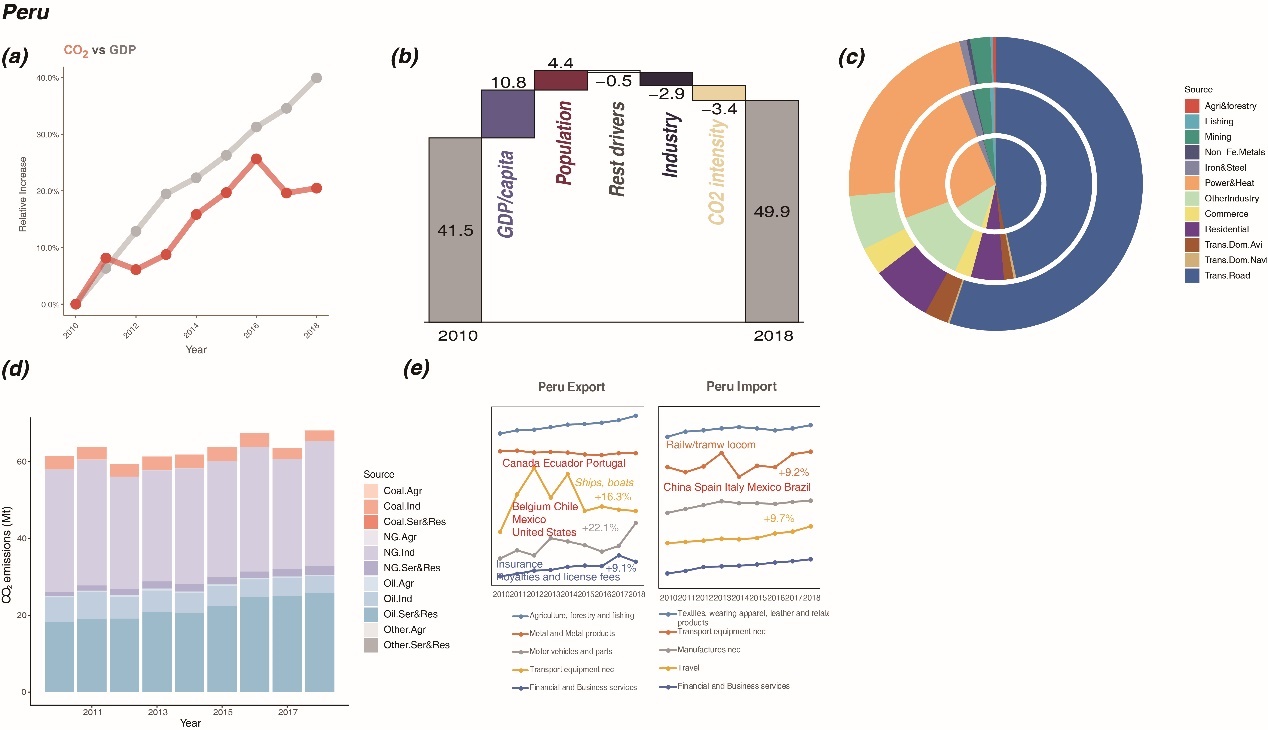


Fig. S12 Relative increase of CO_2_ emissions and GDP (a), emissions change of main drivers (b, in Mt), emissions by sector (c, in 2010, 2014 and 2018 from the inner circle to outer ones), emissions by energy and industry (d), and international trade changes (e) of Myanmar.

CO_2_ emissions under scenarios over 2020-2050

According to our projection, if the emerging emitters follow the 2.5° scenario, the magnitude of emissions from these emerging emitters will strain the limits of the remaining budget of emissions that would avoid 1.5℃ warming[31], leaving very limited carbon space for other countries before 2050, as shown in Fig. S13. Fast carbonization of the 59 emerging emitters would require significant space by 2043 and leaves small room for other countries if the 1.5℃ target is to be met.


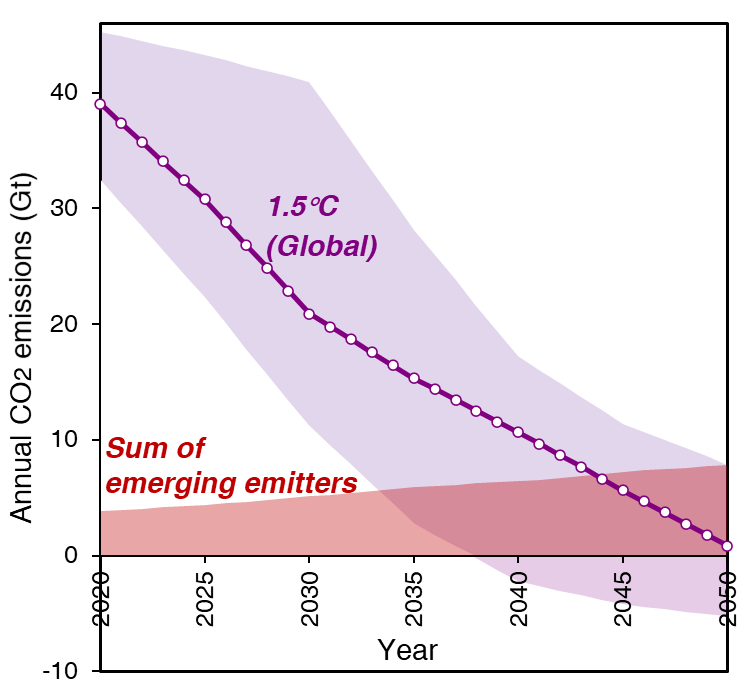


Fig. S13 CO_2_ emissions of emerging emitters over 2020-2050, under the 2.5° scenario.

The comparison between updated data and SSPs data

We selected SSP2 pathway from the GAINS model that inherited CO_2_ emissions results of IIASA MESSAGE model, which usually represents the development along historical patterns. However, GAINS only provides emissions data of major economies but does not cover every country, thus, we further have separated those emerging emitters from the aggregated regions and updated their emission trends in terms of historical data. Although the emerging emitters surge the emissions recently, it is the first time that the future emissions trends of the emerging emitters are updated. Fig. S14 shows the CO_2_ emissions of history, under the 2.5°,2.2°,2.0°and 1.5° scenario, and the downscaled scenarios of SSP1-5 from references[32]. Although the references have country-level data that are compatible with this research, some small countries like Laos are not covered in their downscaling data. Country-specific comparisons between scenarios are provided as follows.


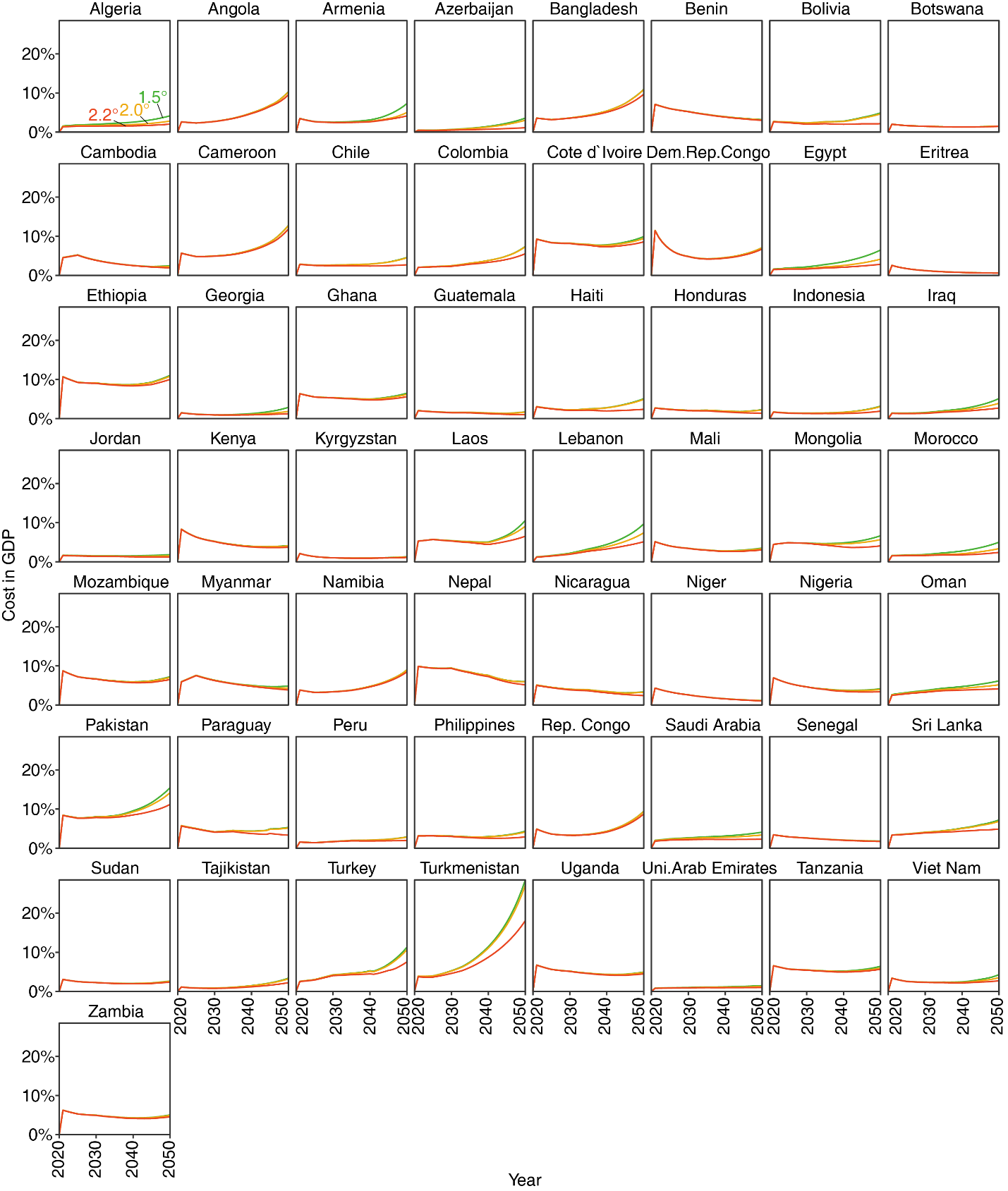


Fig. S14 Annual cost of non-emitting energy deployment under 1.5° (green lines), 2.0° (yellow lines), and 2.2° (red lines) scenarios, in percent GDP. Notes: GDP data of South Sudan and Qatar were unavailable from GAINS GDP by country.


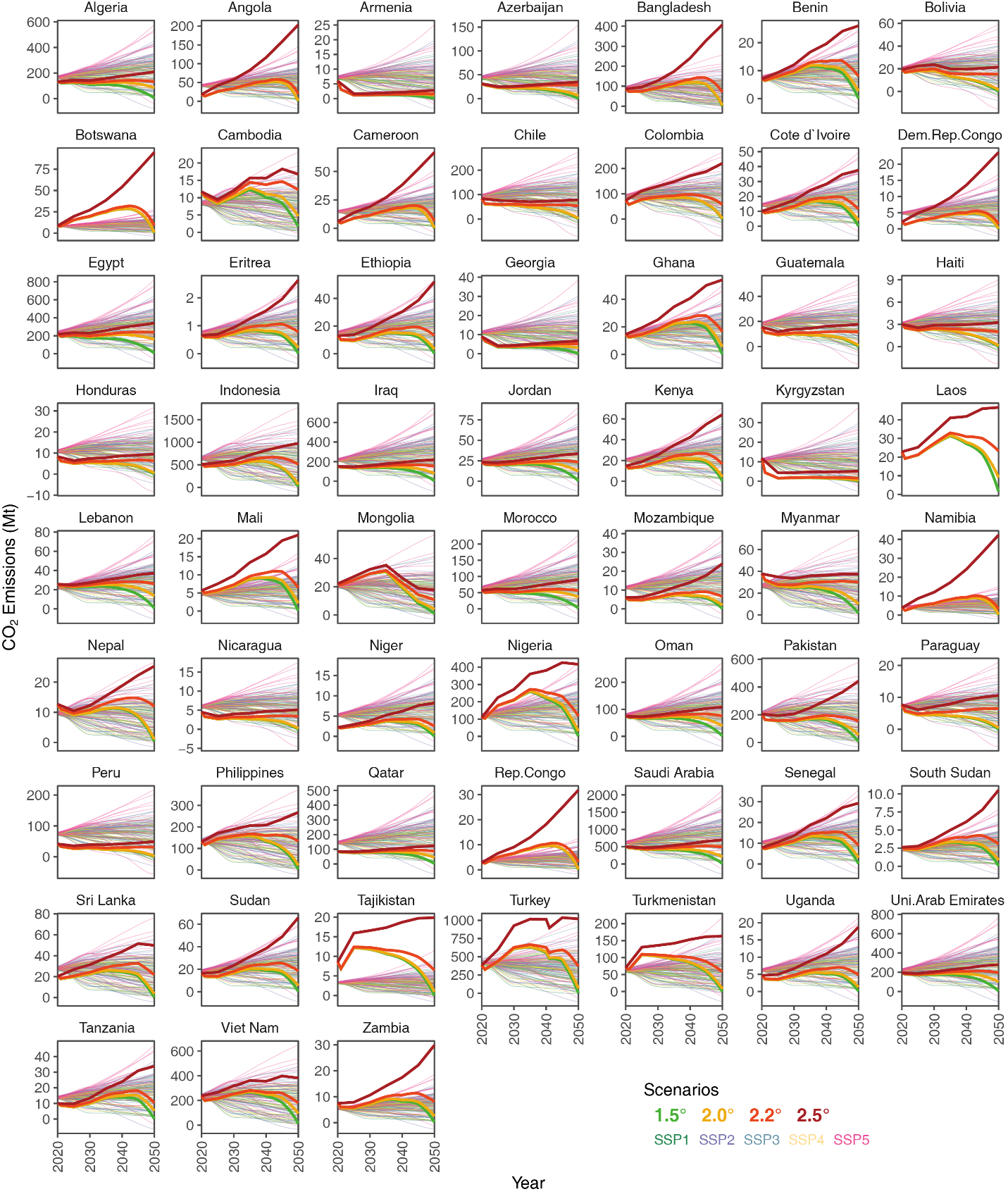


Fig. S15 CO_2_ emissions comparison among scenario sets. The thick lines show the estimations under 2.5°, 2.2°, 2.0°, and 1.5° scenarios, and the thin lines show those from references under SSP1 to SSP5, respectively.

Supplementary Tables

Table. S1 The 59 countries with fast-growing CO_2_ emissions and their developing stage.

Table. S2 CO_2_ emissions of different scenarios over 2020-2050.

DdReferences for Supplementary Information

1. Ang BW. The LMDI approach to decomposition analysis: a practical guide. *Energy Policy* 2005;**33**:867–71.

2. Ang BW. LMDI decomposition approach: A guide for implementation. *Energy Policy* 2015;**86**:233–8.

3. Fricko O, Havlik P, Rogelj J *et al.* The marker quantification of the Shared Socioeconomic Pathway 2: A middle-of-the-road scenario for the 21st century. *Global Environmental Change* 2017;**42**:251–67.

4. International Institute for Applied Systems Analysis. Greenhouse Gas - Air Pollution Interactions and Synergies (GAINS) - IEA WEO 2019 SPS/SDS scenarios. 2020.

5. Amann M, Kiesewetter G, Schöpp W *et al.* Reducing global air pollution: the scope for further policy interventions. *Philosophical Transactions of the Royal Society A: Mathematical, Physical and Engineering Sciences* 2020;**378**:20190331.

6. Rafaj P, Kiesewetter G, Krey V *et al.* Air quality and health implications of 1.5 °C–2 °C climate pathways under considerations of ageing population: a multi-model scenario analysis. *Environ Res Lett* 2021;**16**:045005.

7. Meng J, Way R, Verdolini E *et al.* Comparing expert elicitation and model-based probabilistic technology cost forecasts for the energy transition. *PNAS* 2021;**118**, DOI: 10.1073/pnas.1917165118.

8. International Energy Agency. CO2 emission from fuel combustion. 2019.

9. International Energy Agency. World Energy Balance. 2019.

10. World Bank. World Bank national accounts data. 2020.

11. Le Quéré C, Jackson RB, Jones MW *et al.* Temporary reduction in daily global CO2 emissions during the COVID-19 forced confinement. *Nat Clim Chang* 2020;**10**:647–53.

12. Riahi K, van Vuuren DP, Kriegler E *et al.* The Shared Socioeconomic Pathways and their energy, land use, and greenhouse gas emissions implications: An overview. *Global Environmental Change* 2017;**42**:153–68.

13. KC S, Lutz W. The human core of the shared socioeconomic pathways: Population scenarios by age, sex and level of education for all countries to 2100. *Global Environmental Change* 2017;**42**:181–92.

14. Cuaresma JC. Income projections for climate change research: A framework based on human capital dynamics. *Global Environmental Change* 2017;**42**:226–36.

15. Jiang L, O’Neill BC. Global urbanization projections for the Shared Socioeconomic Pathways. *Global Environmental Change* 2017;**42**:193–9.

16. Leimbach M, Kriegler E, Roming N *et al.* Future growth patterns of world regions – A GDP scenario approach. *Global Environmental Change* 2017;**42**:215–25.

17. Dellink R, Chateau J, Lanzi E *et al.* Long-term economic growth projections in the Shared Socioeconomic Pathways. *Global Environmental Change* 2017;**42**:200–14.

18. Huppmann D, Kriegler E, Krey V *et al.* *IAMC 1.5°C Scenario Explorer and Data Hosted by IIASA*. Integrated Assessment Modeling Consortium & International Institute for Applied Systems Analysis, 2018.

19. Belton B, Filipski M. Rural transformation in central Myanmar: By how much, and for whom? *Journal of Rural Studies* 2019;**67**:166–76.

20. Mondal MAH, Bryan E, Ringler C *et al.* Ethiopian energy status and demand scenarios: Prospects to improve energy efficiency and mitigate GHG emissions. *Energy* 2018;**149**:161–72.

21. Uganda National Ro ads Authority,. *Road Transportation*., 2019.

22. Nabasa, D. The contribution of road transport to the economic development of Uganda. 2018.

23. Electricity Regulation Authority. Install Capacity.

24. Uganda Bureau of Statistics. *Population Projections 2018*., 2019.

25. BACI database. *BACI HS6 REV.1992(1995-2018)*., 2018.

26. Busia. How can Uganda export so much more gold than it mines? Economics. *Economics* 2019.

27. Dun&bradstreet. *Aerospace Products & Parts Manufacturing Companies In Uganda*., 2020.

28. National Statistical Office of Mongolia. *Statistical Yearbook*.

29. Ryu H, Dorjragchaa S, Kim Y *et al.* Electricity-generation mix considering energy security and carbon emission mitigation: Case of Korea and Mongolia. *Energy* 2014;**64**:1071–9.

30. Davaakhuu O, Sharma K, Oczkowski E. Has foreign investment played a role in Mongolia’s export success? *Post-Communist Economies* 2015;**27**:256–67.

31. Allen M, Dube OP, Solecki W *et al.* *Global Warming of 1.5°C. An IPCC Special Report on the Impacts of Global Warming of 1.5°C above Pre-Industrial Levels and Related Global Greenhouse Gas Emission Pathways, in the Context of Strengthening the Global Response to the Threat of Climate Change, Sustainable Development, and Efforts to Eradicate Poverty*., 2018.

32. Gütschow J, Jeffery ML, Günther A *et al.* Country-resolved combined emission and socio-economic pathways based on the Representative Concentration Pathway (RCP) and Shared Socio-Economic Pathway (SSP) scenarios. *Earth System Science Data* 2021;**13**:1005–40.
